# Decoding molecular recognition of inhibitors targeting HDAC2 via molecular dynamics simulations and configurational entropy estimation

**DOI:** 10.1371/journal.pone.0273265

**Published:** 2022-08-18

**Authors:** Suriya Tateing, Nuttee Suree

**Affiliations:** 1 Interdisciplinary Program in Biotechnology, Graduate School, Chiang Mai University, Chiang Mai, Thailand; 2 Division of Biochemistry and Biochemical Innovation, Department of Chemistry, Faculty of Science, Chiang Mai University, Chiang Mai, Thailand; 3 Department of Plant and Soil Sciences, Faculty of Agriculture, Chiang Mai University, Chiang Mai, Thailand; 4 Center of Excellence in Materials Science and Technology, Faculty of Science, Chiang Mai University, Chiang Mai, Thailand; Nippon Medical School, JAPAN

## Abstract

Molecular recognition by enzymes is a complicated process involving thermodynamic energies governing protein-ligand interactions. In order to aid the estimation of inhibitory activity of compounds targeting an enzyme, several computational methods can be employed to dissect this intermolecular contact. Herein, we report a structural dynamics investigation of an epigenetic enzyme HDAC2 in differentiating its binding to various inhibitors within the sub-sites of its active site. Molecular dynamics (MD) simulation was employed to elucidate the intermolecular interactions as well as the dynamics behavior of ligand binding. MD trajectories of five distinct HDAC2-inhibitor complexes reveal that compounds lacking adequate contacts with the opening rim of the active site possess high fluctuation along the cap portion, thus weakening the overall affinity. Key intermolecular interactions determining the effective binding of inhibitors include hydrogen bonds with Gly154, Asp181, and Tyr308; hydrophobic interactions between Phe155/Phe210 and the linker region; and a pi-stacking with Arg39 at the foot pocket. Decomposition of the binding free energy calculated per-residue by MM/PBSA also indicates that the interactions within the internal foot pocket, especially with residues Met35, Leu144, Gly305, and Gly306, can contribute significantly to the ligand binding. Additionally, configurational entropy of the binding was estimated and compared to the scale of the binding free energy in order to assess its contribution to the binding and to differentiate various ligand partners. It was found that the levels of entropic contribution are comparable among a set of structurally similar carbamide ligands, while it is greatly different for the set of unrelated ligands, ranging from 2.75 to 16.38 kcal/mol for the five inhibitors examined. These findings exemplify the importance of assessing molecular dynamics as well as estimating the entropic contribution in evaluating the ligand binding mechanism.

## Introduction

Several physiological functions and gene expressions are epigenetically controlled through the modification of chromatin structures and the alteration of histone acetylation and deacetylation levels in the nucleosomes [1–3]. Antagonistic enzymes involved in these processes include histone acetyltransferases (HATs) and histone deacetylases (HDACs). For histone acetylation, HATs acetylate the ε-NH_2_ group of histone lysines, thereby neutralizing the charge on the protein surface, relaxing the chromatin structure, and thus allowing the access of transcription factors [4–7]. HDACs, on the other hand, deacetylate the histone lysine residues and reconstitute the positive charge, thus compacting the chromatin structure and preventing the gene expression from occurring [5, 6]. This important role in gene expression makes HDACs critical targets for drug development, as their dysfunction and inhibition have been associated with several diseases, including cancer [7–10], neurological, renal, lung, and cardiovascular diseases [11–16], as well as inflammatory immune disease [17–19].

HDAC enzymes can be subcategorized into four families based upon their structure and function, denoted as classes I, II, III, and IV HDACs [20]. HDAC2, a member of the class I HDAC family, is one of the most studied isoforms and it possesses a high enantioselectivity towards its substrates [21]. Aside from its structural homology and catalytic activity similar to other isoforms within the class I HDACs, HDAC2 also shares a highly conserved catalytic domain that comprises a lipophilic tube, a deep catalytic site where its Zn^2+^ cofactor is located, and an internal ‘foot pocket’ [7]. The lipophilic tube connecting the opening rim to the catalytic site is formed by Gly154, Phe155, His183, Phe210 and Leu276 [22, 23]. The catalytic site is surrounded by Asp181, His183, and Asp269, while the internal ‘foot pocket’ is formed by Tyr29, Met35, Arg39, Phe114, and Leu144 residues [22–24]. This elongated shape of the substrate binding site also provides high specificity to HDAC2 inhibitors, which usually contain a cap group, a hydrophobic linker portion, and a zinc-binding group (ZBG) [25–27]. In recent years, various novel HDAC inhibitors with *in vitro* and *in vivo* potencies have been reported for their potential therapeutic effects against chromatin-related diseases [28, 29].

In order to help accelerate the process of inhibitor development, computational methods are generally employed in order to dissect the molecular recognition by which the target protein confers activity, thus providing a powerful tool to virtually search for candidate compounds that potentially bind tightly to the binding site. Simulation of molecular motions and structural information of the binding at atomic details have also helped elucidate the thermodynamic energy terms involved in the intermolecular recognition [30].

Molecular dynamics (MD) simulation and molecular mechanics (MM) calculations have been used in the designing of HDAC2 inhibitors, especially focusing on the non-bonding energy contributed by the binding with the composite active site (CAS) [31]. Dewaker, et al. demonstrated the use of energy decomposition of residues contacting the ligand cap group, the ZBG, and the foot-pocket residues in the design of new inhibitor compounds potentially bind more effectively to the active site. However, a comparison between the calculated binding energy and the actual experimental data, e.g. IC_50_ values of the ligands, is still necessary in order to derive a more reliable binding mechanism. Additionally, although MD simulation and MM calculations could help estimate the enthalpic contribution of the binding, the role of the entropic contribution and its approximation have remained elusive in many molecular systems. One of the prime interests here is the configurational entropy which is caused by the variation of conformational states of the protein. Currently, several methods have been devised in order to calculate entropy. One example is an empirical method that subcategorizes entropic contribution into two parts: solvation free entropy and configurational free entropy [32, 33]. The quasi-harmonic approximation (QH) is another method introduced by Karplus and Kushick in 1981 [34] that estimates the configurational entropy based upon MD simulation by building a Gaussian distribution of protein conformations [35].

For most applications of MD simulation in biomolecular systems, entropic contributions to the binding free energy are generally neglected due to their high computational cost or complicated estimation processes. Nonetheless, insight into this particular energetic contribution could provide deeper understanding of the molecular system of interest since entropy can have different levels of impact on molecular recognition. For HDACs, the Christianson group has reported the role of entropy in driving selectivity for inhibitors targeting a class-IIb HDAC6 vs. a class-I HDAC8 enzyme [36]. Isothermal titration calorimetry experiments revealed that the binding entropy of HDAC6-specific inhibitors was more favorable for HDAC6 than for HDAC8 [36]. This difference in entropic contribution was believed to stem from differences in configurational entropy and/or desolvation entropy, particularly through the interactions with the aromatic cleft of the binding site [36].

In this study, we aimed to investigate a range of binding mechanisms and molecular dynamics properties involved in the ligand binding of HDAC2 in order to appraise the influential factors contributing to the inhibitory activity of various ligands. MD simulations of complexes formed between HDAC2 and several known inhibitors were performed and analyzed for the modes of binding in the sub-sites of its active site cleft. Molecular mechanics Poisson-Boltzmann Surface Area (MM/PBSA) calculations were also employed to estimate the energetic contributions to the inhibitor binding as well as to investigate the level of influential interaction of each important amino acid residue. Additionally, we quantified the entropic contribution to the overall binding energy, and made comparisons among the structurally diverse and similar ligands. The data from this study provide new insight into the significance of dynamics behavior and an entropic contribution in delineating inhibitor binding mechanisms. This approach could become a powerful tool in selecting and optimizing novel ligand candidates for HDACs.

## Material and methods

### Protein-ligand complex preparation

Crystal structures of human HDAC2 in complex with various inhibitors were retrieved from the Protein Data Bank [37, 38], which include complexes with either N-(2-aminophenyl)benzamide [22] (LLX, PDB ID: 3MAX, 2.05 Å resolution), octanedioic acid hydroxyamide phenylamide [39] (SAHA or vorinostat, PDB ID: 4LXZ, 1.85 Å resolution), 4-(acetylamino)-N-[2-amino-5-(thiophen-2-yl)phenyl]benzamide [39] (20Y, PDB ID: 4LY1, 1.57 Å resolution), N-(4-amino-4’-fluoro[1,1’-biphenyl]-3-yl)oxane-4-carboxamide [40] (IWX or BRD4884, PDB ID: 5IWG, 1.66 Å resolution), or (3-exo)-N-(4-amino-4’-fluoro[1,1’-biphenyl]-3-yl)-8-oxabicyclo[3.2.1]octane-3-carboxamide [40] (6EZ or BRD7232, PDB ID: 5IX0, 1.72 Å resolution). The five crystal structure complexes were prepared as input coordinate files for the molecular dynamics simulations using a combination software involving antechamber and LEaP programs. Atomic partial charges of inhibitors were obtained by fitting the electrostatic potentials (ESP), derived using Gaussian09 (G09), with HF/6-31G* as the basis set [41]. The protonation configurations of the titratable histidine residues were predicted by the APBS-PDB2PQR server [42]. The webserver was employed to predict the proper protonation states for each titratable residue, which results in relevant output files pqr, stdout.txt, and stderr.txt. Especially, the protonation states of HIS to be HID or HIE of protein were displayed in the pqr output file, while the calculated pKa values were shown in the log output file. Notably, the mass and charge of the metal ion (Zn^2+^) were assigned via a parameter file frcmod.ions234lm_126_tip3p (for divalent ions for the TIP3P water model), which yielded the mass and charge of Zn^2+^ of 65.4 and 2.0, respectively. The results are summarized in S1 Table of S1 File. For the following molecular mechanics (MM) minimizations and MD simulations, AMBER14SB force field [43, 44] and the general AMBER force field [45] (gaff) were employed to establish the potential of proteins and inhibitors, respectively. Water molecules were randomly replaced by chloride ions in order to neutralize the charge of the system. Finally, the entire system was solvated in a cubic periodic box of the TIP3P explicit solvent model [46], keeping the distances between the edges of the water box and the closest atom of the solutes at least 10 Å. To avoid any edge effects, periodic boundary conditions were applied during the entire MD simulations. All HDAC2-inhibitor complex systems were converted via a ParmED script to be compatible with the Gromacs format.

### Molecular dynamics simulations

For each molecular system, energy minimization and MD simulations were performed using Gromacs 6.4.3 package [47]. For the HDAC2-inhibitor complexes, the protein-ligand modeling was performed using the LEaP from AmberTools20 [48]. Subsequently, for the force fields employed, leaprc.protein.ff14SB [38], leaprc.water.tip3p, and leaprc.gaff were sourced for protein, water, and ligand, respectively, which would load a frcmod.ions234lm_126_tip3p file that has a parameter set for Zn^2+^ ion for the TIP3P water model. This command also loaded an AMBER-format parameter set file and placed it in the variable ‘frcmod.ionsjc_tip3p’ that consists of monovalent ion parameters and a TIP3P water model. The force field parameter for Zn^2+^ ion adopted in our simulations were designed by Li and Merz [49] and employed according to the Amber 2020 protocol. The AMBER topology files were converted to Gromacs topology files via ParmED script. Each system was subjected to an energy minimization without restriction, which entailed a 5000-step steepest descent minimization. For the equilibration process, a position restraint was applied to the protein and the bound inhibitor both for NVT and NPT ensembles. Particle Mesh Ewald [49] algorithm [50, 51] was employed to handle the long-range electrostatic interactions. The cutoff distance for the van der Waals energy interactions was set at 14 Å and the LINCS algorithm for bond constraints was used [52]. The systems were gradually heated in the NVT ensemble from 0 to 300 K for over 100 ps. Subsequently, an isothermal isobaric ensemble (NPT) with periodic boundary conditions was performed for 100 ps. Finally, 100-ns MD simulations were carried out to extend the MD production for each system in an isothermal isobaric ensemble (NPT) [53] using periodic boundary conditions. The SHAKE method [54] was applied to constrain all covalent bonds involving hydrogen atoms. Each simulation was coupled to a 300 K thermal bath at 1.0 bar by applying Berendsen the thermostat and the Parrinello-Rahman barostat, respectively. The temperature and pressure coupling parameters were separately treated for more accuracy. During the sampling process, the coordinates were saved every 0.1 ps and the conformations generated from the simulations were used for further MD trajectory analyses. MD trajectories and structures of each system were visualized using Visual Molecular Dynamics [55] software [55] and Accelrys Discovery Studio Visualizer 4.0 (Accelrys Software Inc.). SigmaPlot 12.5 (Systat Software, San Jose, CA) was used for generating all plots of the various parameters.

Coordination number of the Zn^2+^ cofactor ion was calculated according to the concept of quantifying atomic interactions surrounding the central metal atom [56, 57] using the following equation:

$$n=\int_{R_{1}}^{R_{2}}4\pi r^{2}\rho g(r)dr$$ (1),

where *n* is the number of specific atoms in a peak of radius distribution function (RDF), *r* is the distance from the atom to the target metal atom, R_1_ is the starting position (at 0 nm), R_2_ is the position of the first shell (with a cutoff distance of 0.27 nm), *ρ* is the number density of the atom in the main phase, and g(*r*) is the RDFs from the atom to the target atom.

Additionally, coordination of the Zn^2+^ metal cofactor was visualized from the MD simulations using the Accelrys Discovery Studio Visualizer. Briefly, from the MD trajectories of all complexes, eleven snapshots representing every 10 ns were extracted from the runs and superimposed to visualize the coordination number of the cofactor ion. Atomic distances between the Zn^2+^ cofactor of HDAC2 and a nearby heavy atom of amino acid residues or ligands were also measured.

### Principal component analysis

Trajectories of the MD simulations were utilized to identify the concerted motions of HDAC2-ligand complexes. For the estimation of configurational entropy change in each protein-ligand complex, calculations were based upon the MD trajectories of the apo-protein, individual ligands, as well as the protein-ligand complexes. The last snapshots (80–100 ns) of MD trajectories of apo-proteins, ligands, or protein-ligand complexes were acquired. First, for constructing the covariance matrix, translational and rotational movements of the HDAC2 protein were removed. To do this, a command g_trjconv, a utility within the Gromacs suite, was employed. This command contains a command -fit rot+trans as an additional function to systematically remove rotation and translation motions by aligning the Cα atoms of structures within a single MD run to those Cα atoms of the initial structure. Of note, for the entropy calculation, the initial structure was the structure at 80 ns (as the range of calculation was 80–100 ns). However, for the density plot visualization, the initial structure was the structure at 10 ns (as the range for this observation was 10–100 ns).

The covariance matrix was calculated using Cartesian coordinates of the Cα atoms of the protein. This method was also applied for the inhibitor ligands using all of their atoms. Principal component analysis (PCA) was employed for estimating the direction of molecular motions based upon the covariance matrix of the atomic fluctuations. Diagonalization of this matrix yields a set of eigenvectors and eigenvalues that describe collective modes of fluctuations of the molecules. Eigenvectors represent the direction of the motions, whereas the corresponding eigenvalues represent the amplitudes of those directions along the multidimensional space [58]. Displacement of atoms along each eigenvector represents concerted motions of the protein along each direction. Gromacs utilities, including ‘g_covar’ and ‘g_anaeig’, were used to solve some functionally relevant motions. Particularly, the ‘g_covar’ tool was used to perform the diagonalization of the computation elements from the covariance matrix of positional fluctuations found within the Cα carbon atoms of the protein molecules. The ‘g_anaeig’ tool was used to analyze the free energy landscape for the projections along the two eigenvectors, and the entropy term was then estimated by the quasi-harmonic approximation (QH) method. Differences between the entropies calculated in the bound and unbound forms of the protein and ligands were used to calculate the configurational entropy change.

### Molecular mechanics calculations

For HDAC2-inhibitors complexes, 1,000 snapshots taken from the last 10 ns trajectories were obtained for calculating the binding free energy using the molecular mechanics Poisson-Boltzmann Surface Area method (g_mmpbsa) [59], as well as the APBS software suite [42], which is implemented in the Gromacs package. The binding free energy was calculated using Eq (2).

$${\Delta G}_{\mathrm{binding}}={\Delta G}_{\mathrm{complex}}-({\Delta G}_{\mathrm{protein}}+{\Delta G}_{\mathrm{ligand}})$$ (2)

$${\Delta G}_{\mathrm{binding}}={\Delta E}_{\mathrm{MM}}+{\Delta G}_{\mathrm{solvation}}-T\Delta S$$ (3)

$${\Delta E}_{\mathrm{MM}}={\Delta E}_{\mathrm{bonded}}+{\Delta E}_{\mathrm{vdW}}+{\Delta E}_{\mathrm{elec}}$$ (4)

$${\Delta G}_{\mathrm{solvation}}={\Delta G}_{\mathrm{polar}}+{\Delta G}_{\mathrm{nonpolar}}$$ (5)

$${\Delta G}_{\mathrm{nonpolar}}=\gamma \times \mathrm{SASA}+b$$ (6),

where Δ*G*_**binding**_ is an estimate of the binding free energy between the HDAC protein and the ligand. Each free energy term in Eq (**2**) is a sum of the gas phase molecular mechanical energy (*ΔE*_**MM**_), the solvation free energy (*ΔG*_**solvation**_) and the entropic term (*−TΔS*). In Eq (4), *ΔE*_**MM**_ can be divided into contributions from the bonded term (*ΔE*_**bonded**_), which includes bond, angle, and dihedral terms, and two non-bonded terms, which are the van der Waals energy (*ΔE*_**vdW**_) and the electrostatic energy (*ΔE*_**elec**_) in the gas phase. The solvation free energy (*ΔG*_**solvation**_) was estimated according to Eq (5) using continuum solvent methods and it can be divided into two parts: the polar contribution (*ΔG*_**polar**_) and the nonpolar contribution (*ΔG*_**nonpolar**_). The electrostatic solvation energy was determined using the finite difference PB model. The dielectric constants used for the interior [59] and the exterior (water) were 1 and 80, respectively. Atomic radii and charges were the same as those used in the MD simulations. The nonpolar contribution (*ΔG*_**nonpolar**_) to the solvation free energy was calculated from the solvent-accessible surface area [59], which is expressed in Eq (6). The standard numerical values of *γ* is 0.00542 kcal/(mol·Å^**2**^), and *β* is 0.92 kcal/mol for the Poisson-Boltzmann method. The probe radius of the solvent was set to 1.4 Å.

## Results and discussions

### Molecular dynamics simulations of the HDAC2-inhibitors complexes

In order to gain a better perspective on the ligand binding of HDAC2 as well as the corresponding changes in their dynamics behavior, MD simulations were performed on the crystal structures of complexes involving HDAC2 and five known inhibitors. These selected ligands, which include LLX, SAHA, 20Y, IWX, or 6EZ, possess distinct structural scaffolds, and their reported inhibitory activities (IC_50_) also range from 1.7 nM to 1,200 nM. Initially, coordinates from the crystal structures were used as input for our 100-ns MD simulations, after which the resulting dynamics parameters, as well as the three-dimensional spatial snapshots, were acquired and analyzed. In this particular study we focused on the structural motions observed within the active site cleft of HDAC2 and its neighboring areas, that could shed light onto the mechanisms by which the selected ligands convey various levels of inhibitory activity. Significant changes in dynamics and energetic contributions of these residues when interacting with the ligands could also imply their important roles in conferring selectivity for suitable ligands.

**Fig 1:**
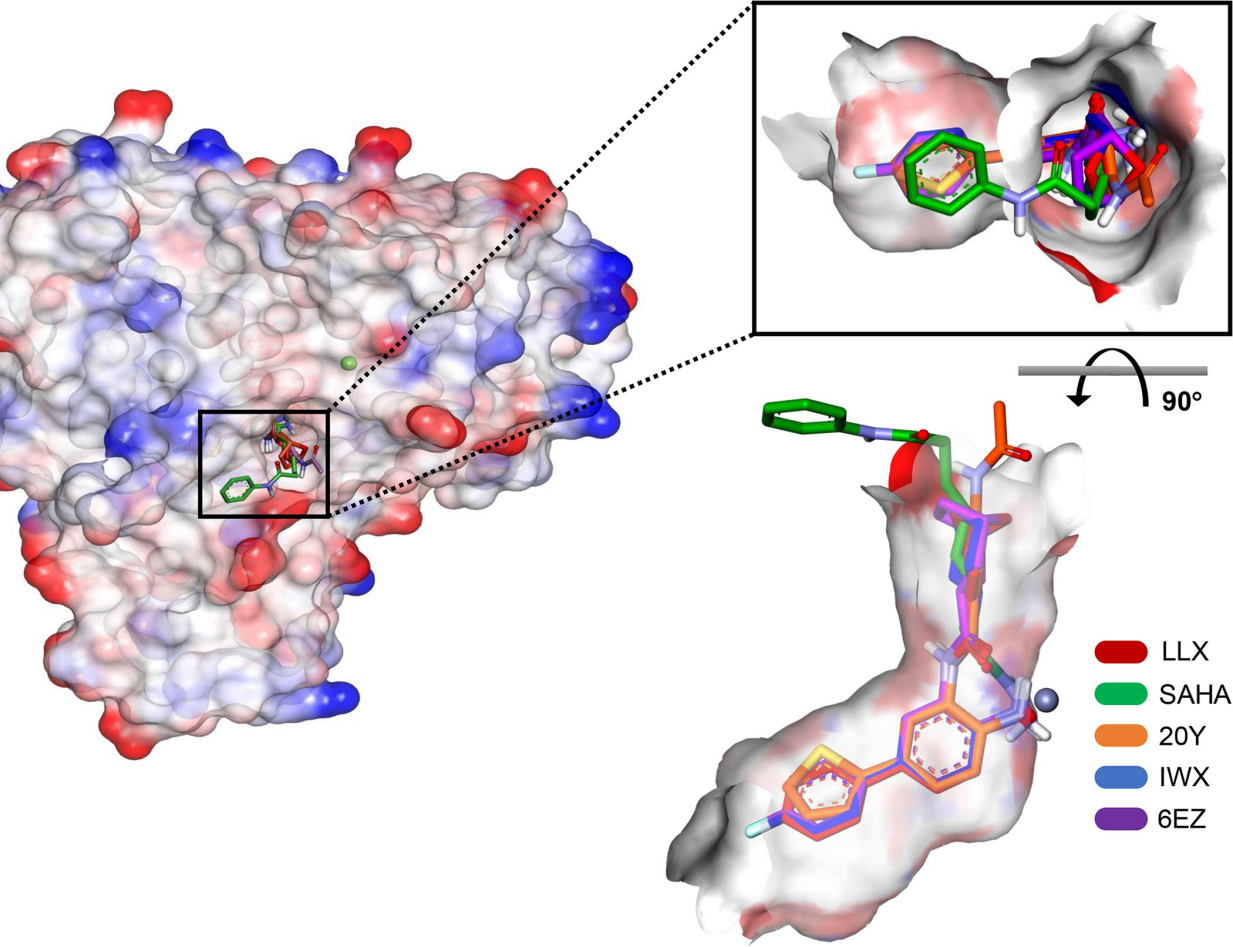
Structural overview of HDAC2 and its active site location. (Left) Electrostatic surface representation of HDAC2 showing the binding location of its inhibitors. (Right) Overlaid structures of inhibitor ligands when bound to the active site. The inhibitor molecules are illustrated in stick representations showing stabilized conformations of LLX (red), SAHA [60], 20Y (orange), IWX (blue), and 6EZ (purple) ligands, which are structurally overlaid using heavy atom coordinates. Zinc ion is shown as a gray ball.

Fig 1 depicts an overview of how the five ligands were nested within the active site pocket. HDAC2 contains a lipophilic tube that links the opening surface to the catalytic triad (His145, His146, and Tyr308), and the adjacent Zn^2+^ cofactor binding region (Asp181, His183, and Asp269). Deep inside the cleft also lies an internal cavity or a ‘foot pocket’, which is formed by Tyr29, Met35, Arg39, Phe114, and Leu144, which could convey additional specificity to the ligand binding. As observed from the overlaid crystal structures, ligands that contain thin and elongated shapes could thus be threaded into the pocket and can extend their binding interactions, surpassing the cofactor binding area to this deeper site. The foot pocket of the active site cleft generally contains multiple water molecules, which are commonly found within crystal structures of complexes with smaller-sized ligands [22]. Additionally, the opening rim of the active site cleft could also provide additional interactions to augment the ligand affinity and specificity. SAHA is a good example of ligands that take advantage of this binding mechanism since its aniline group partially acts as a ‘knob’ plugging the opening region. From these different regions of binding, it is plausible that any ligand that employs all areas of intermolecular interactions would create a stable complex, exhibiting a more stable dynamics pattern, and potentially contributing to stronger inhibitory activity.

**Fig 2:**
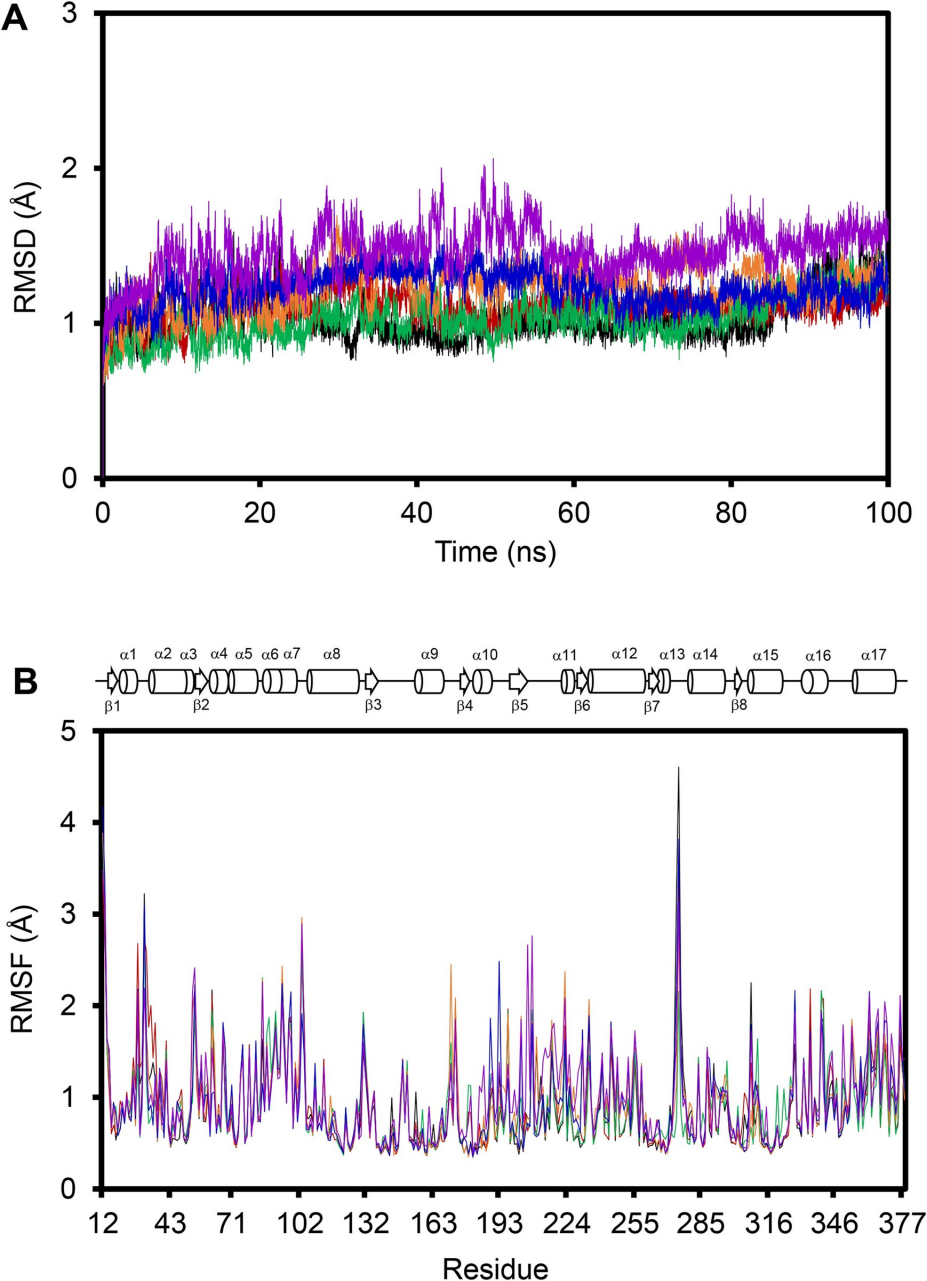
(A) Root-mean-square deviations (RMSD) and (B) root-mean-square fluctuations (RMSF) of backbone atoms on HDAC2 when bound or unbound by inhibitors. Unbound HDAC2 (black) is compared with its complex forms with LLX (red), SAHA [60], 20Y (orange), IWX (blue), and 6EZ (purple). Topology diagram of HDAC2 is also illustrated atop of the RMSF chart to indicate range of secondary structures along the residue sequence.

To delineate the binding mechanism in a more comprehensive manner, MD simulations were employed to analyze changes in motions observed within the HDAC protein upon ligand binding. Fig 2 shows the dynamics parameters collected from our 100-ns MD simulations of the protein-ligand complexes. Simulation equilibria were evaluated by comparing the root-mean-square deviation (RMSD) of the backbone atoms relative to the initial coordinates (Fig 2A). Additionally, the MD simulations of the unbound HDAC2 and all complexes were repeated to observe the ranges of fluctuation comparing between the runs and throughout the 100-ns simulation period in order to justify if the simulation time used was sufficient. It was found that the RMSD levels fluctuated within a range of 1 Å (S1 Fig in S1 File). An extended MD run of HDAC2-20Y complex also showed similar range of fluctuation (S2 Fig in S1 File). Overlay of snapshots of the complex structures obtained at 100 ns and at 200 ns of the MD simulation shows similar conformations, with an RMSD between the heavy atoms of the two structures of 0.8187 Å (S3 Fig in S1 File). Thus, we selected 100 ns as the simulation time for this study. It is apparent that the RMSDs of all the ligand complexes fluctuate in slightly different ranges. The highest fluctuations were observed for the complexes of HDAC2-6EZ (purple) and HDAC2-20Y (orange), especially during the first 60 ns of simulation. On the other hand, the HDAC2-IWX complex (blue) fluctuates the least during the entire simulation. Nevertheless, all molecular systems reached a relatively steady period after 80 ns, indicating that the 100-ns simulation duration is sufficient for a comparative study. However, the information from the RMSD data might not be adequate to describe localized motions of the protein structures. Therefore, root-mean-square fluctuations (RMSF) data were also collected and compared among all complexes as well as with the free enzyme.

### Residual structural fluctuations of the bound ligands

Fig 2B shows spatial fluctuations of the backbone heavy atoms of all residues along the protein sequence, comparing the free protein with the protein-ligand complexes. As anticipated, the regions with most fluctuation are located among the loops connecting secondary structure motifs within the protein, particularly at the α13/α14 loop that exhibits the highest fluctuation. However, when focusing only on the active-site residues (His145, His146, Asp181, His183, Asp269, and Tyr308) or the internal cavity residues (Tyr29, Met35, Arg39, Phe114, Leu144, Gly305, and Gly306), fluctuations among these regions do not significantly differ, and no obvious trend could thus be derived from the different ligand complexes.

**Fig 3:**
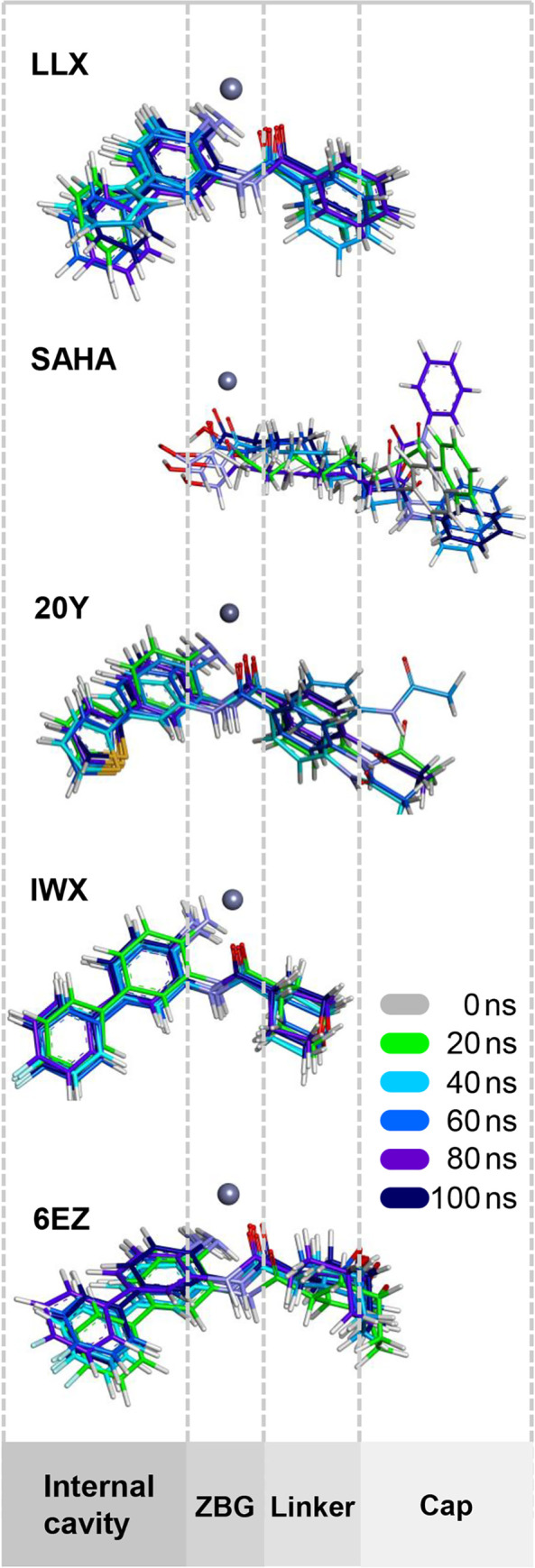
Structural overlays of ligand conformations observed at different time points during the 100-ns MD runs. Relative positioning of the bound ligand structures along the active site canal is also indicated with dashed lines. Ligand positions are aligned using the coordinates of the Zn^2+^ cofactors, shown as gray spheres, as a reference point. ZBG denotes the ligand’s zinc-binding group.

Since the observed spatial changes within the protein structure did not yield any complete differentiation among the ligands, we then asked whether there could be any differences observable within the ligand molecules, possibly over various stages of time. To this end, snapshots of all ligand structures taken from the different time points of simulation, including 0, 20, 40, 60, 80, and 100 ns, were overlaid and compared for their molecular motions. As illustrated in Fig 3, all five ligands have different levels of movement relative to the active-site location. When mapped onto the estimated position of ligand binding, it is apparent that LLX, 20Y, IWX, and 6EZ insert themselves deep inside the pocket and reach the internal cavity. SAHA, on the other hand, extends to only the cofactor binding region but uses its aniline moiety as a cap group in order to interact with the opening surface. This pattern of cap binding at the opening region can also be seen, though to a lesser extent, in the binding of 20Y, 6EZ, and LLX, but not in the case of IWX. However, it is apparent that the cap portion of the ligands may not have sufficient intermolecular interaction to restrict the overhang structures as they fluctuate highly in the region protruding from the active-site canal.

### Intermolecular interactions restraining the ligand binding

**Fig 4:**
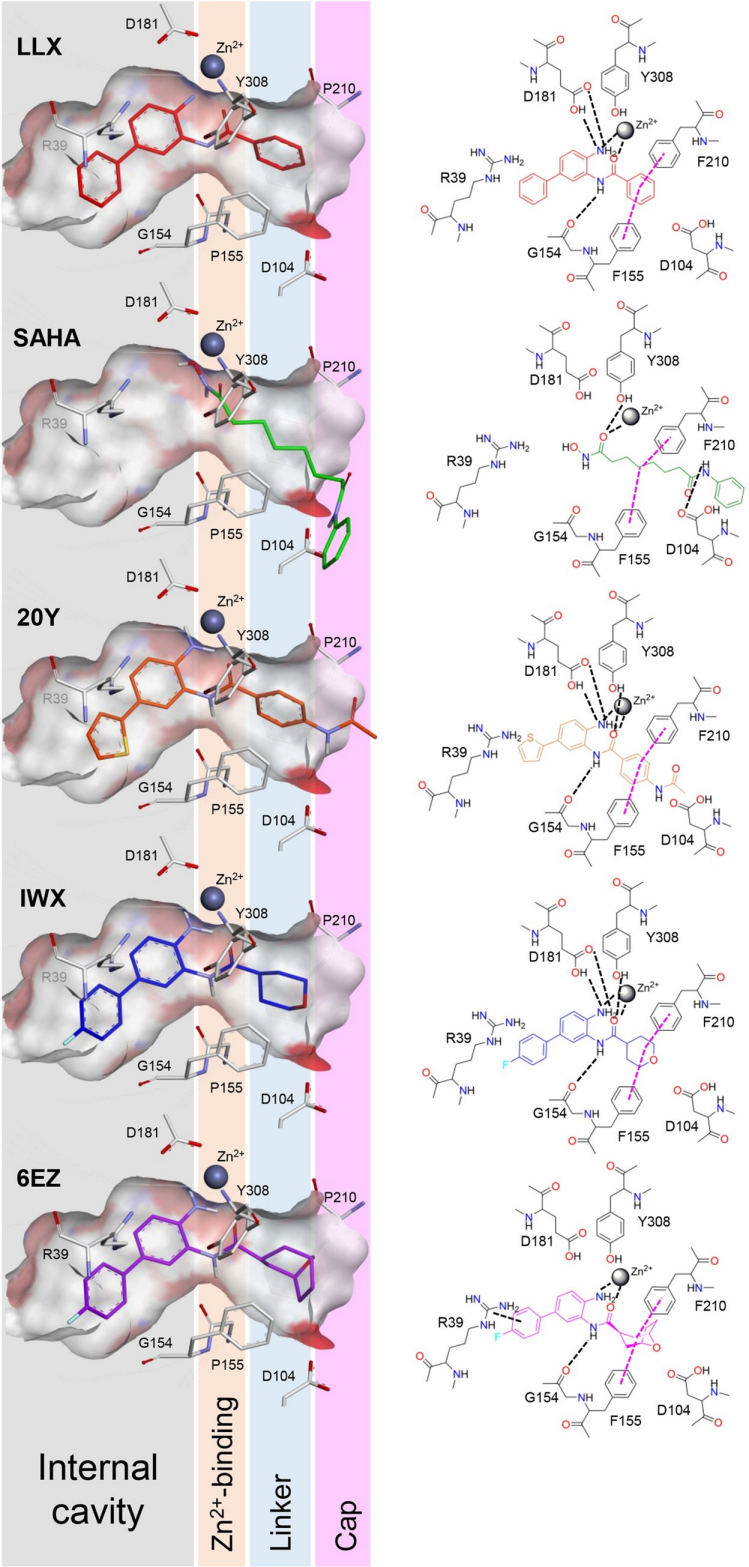
Binding interactions between inhibitor ligands and the interacting amino acid residues lining the active site of HDAC2. (Left) three-dimensional representations of the ligands when nested inside the active site canal. Inhibitor ligands LLX (red), SAHA [60], 20Y (orange), IWX (blue), and 6EZ (purple) are shown in stick representations. Zn^2+^ cofactors are shown as gray spheres. Positions of the ligands along the active site are compared for their structural regions of internal-cavity binding, Zn^2+^ binding, linkers, and cap groups. (Right) two-dimensional diagrams summarizing the protein-ligand binding interactions. Hydrogen bonding networks and cofactor coordination are highlighted in black dashed lines, whereas hydrophobic interactions are indicated with purple dashed lines.

Key binding interactions between the active site residues and the ligands are also summarized in Fig 4. Although many hydrophobic interactions collectively contribute to binding affinity, a few hydrogen bonds are prominently found within the active site, particularly near the catalytic triad and the cofactor binding center. These include the backbone carbonyl of Gly154 and the side-chain carboxyl of Asp181, which is also a part of the Zn^2+^-binding residues (Asp181, His183, and Asp269), and the side-chain hydroxyl of Tyr308. Additionally, histidine residues located near the catalytic triad, His145 and His146, can form hydrogen bonds with the ligands, particularly at the hydroxyl or the amine moieties of the ligand structures. These interactions near the Zn^2+^ cofactor could further anchor the inhibitors to remain within the active site (S4 Fig in S1 File). For the ligands with high inhibitory potencies (LLX, 20Y, and IWX, exhibiting IC_50_ values of 27, 56.3, and 62 nM, respectively) (S3 and S5 Tables in S1 File), two or three of these hydrogen bonds are observable within the complexes. On the other hand, ligands with lower levels of potency (SAHA and 6EZ, exhibiting IC_50_ values of 251 and 168 nM, respectively) form only one hydrogen bond. The correlation between the number of hydrogen bonds formed and the inhibitor potency level coincides with several studies [61–64] suggesting the role of hydrogen bonds as one of the key indicators for a successful inhibitor. Of note, the reported IC_50_ values for HDAC2 inhibitors can be varied based upon different studies. Therefore, an extensive literature review of IC_50_ values was conducted and summarized in S5 Table of S1 File. Values selected for use in our calculation are the results from the most similar methods of determination. Additionally, we did not convert the IC_50_ values into *K*_i_ due to the lack of the information of substrate concentration and *K*_m_, which were not explicitly stated in most studies reported.

Notably, a moderate level of hydrophobic interactions at the opening surface of the active site cleft are also identified, namely with aromatic rings of Phe155 and Phe210. These two residues interact with the linker portion of the ligand structures and can provide particular pi-pi stabilization for the ligands that contain aromatic moieties in this region such as LLX and 20Y, both of which exhibit higher levels of potency. At the deeper side at the foot pocket, Arg39 provides an additional cation-pi intermolecular interaction further anchoring the ligands reaching this site. All the ligands, except SAHA, contain an aromatic moiety at this particular location. The role of Arg39 contributing to the ligand affinity observed here agrees well with previous studies [29, 65, 66]. For the Zn^2+^metal chelation, key residues Asp181, His183, and Asp269 predominantly act as chelators for the Zn^2+^ cofactor. In the absence of ligand, a water molecule fulfills the coordination of Zn^2+^. For ligand binding, however, this water molecule is replaced by a carbonyl oxygen atom from the ligand inhibitor participating in the chelation (S4 Fig in S1 File) and the coordination number of Zn^2+^ remained the same. Atomic distance ranges of metal chelation are summarized in S5 Fig and S2 Table of S1 File. For the calculation of coordination number of the Zn^2+^ cofactor ion, the number of specific atoms in a peak of radius distribution function (RDF) was computed according to Eq (1) via a command g_rdf, a utility function implemented in the GROMACS package. The initial calculation resulted in distributions of atomic partners around the ion, as illustrated in S6 Fig of S1 File. It was observed that the distance trends of the first peaks, which indicate the first hydration shell, were similar among the complexes and centered around 0.2 nm. To distinguish between the first and the second hydration shells, a cutoff distance was placed at 0.27 nm. Since the coordination of the ion is mainly contributed by the first shell, coordination numbers can be calculated from the integral function. The integration yielded an overall running coordination number for the Zn^2+^ ion as a result of interactions with its atomic partners lining the catalytic site of HDAC2 (S7 Fig in S1 File). From the entire 100-ns MD simulations, the calculated coordination numbers of the Zn^2+^ were 6.00, 5.86, 5.29, 5.13, and 5.01 for the complexes between HDAC2 and LLX, SAHA, 20Y, IWX, and 6EZ, respectively. Of note, these coordination numbers were determined using the same set of the R_1_ and R_2_ values for Eq (1).

Notably, the atomic partners coordinating the Zn^2+^ ion are different between the event of substrate catalysis of HDAC2 and the event of inhibitor binding. HDACs employ a promoted-water mechanism for catalysis, in which the active site metal ion (Zn^2+^) and a histidine general base activate a metal-bound water molecule for the nucleophilic attack of the native substrate [67]. For the inhibitor binding, however, HDAC inhibitors often exploit the chelation interactions with the catalytic Zn^2+^ ion, thus replacing this water molecule from the active site. For example, hydroxamate and benzamide derivatives ionize to form exceedingly stable 5- and 6-membered rings that efficiently chelate the Zn^2+^ [68].

Taken together, it can be summarized that the key intermolecular interactions may include: 1) hydrogen bonds with His145, His146, Gly154, Asp181, and Tyr308; 2) hydrophobic interactions between Phe155/Phe210 and the linker region; 3) a pi-stacking with the side chain of Arg39 at the foot pocket and 4) metal chelation contributions from the ligand and the Zn^2+^-binding residues (Asp181, His183, and Asp269). Different combinations of these key interactions could provide a spectrum of ligand affinities and thus inhibitory activities. They could also be perceived as determining factors to predict the inhibitory potency of a ligand. For example, 6EZ that interacts with both the foot pocket and the linker regions, but forms only one hydrogen bond with the active site, exhibits only a moderate level of potency (IC_50_ = 168 nM) when compared to other ligands that have robust interactions with the foot pocket. On the other hand, LLX that forms three hydrogen bonds with D181 and G154 and interacts with both the foot pocket and the linker regions, exhibits a higher level of inhibitory potency (IC_50_ = 27 nM).

### Binding energetic contribution by the active-site residues

**Fig 5:**
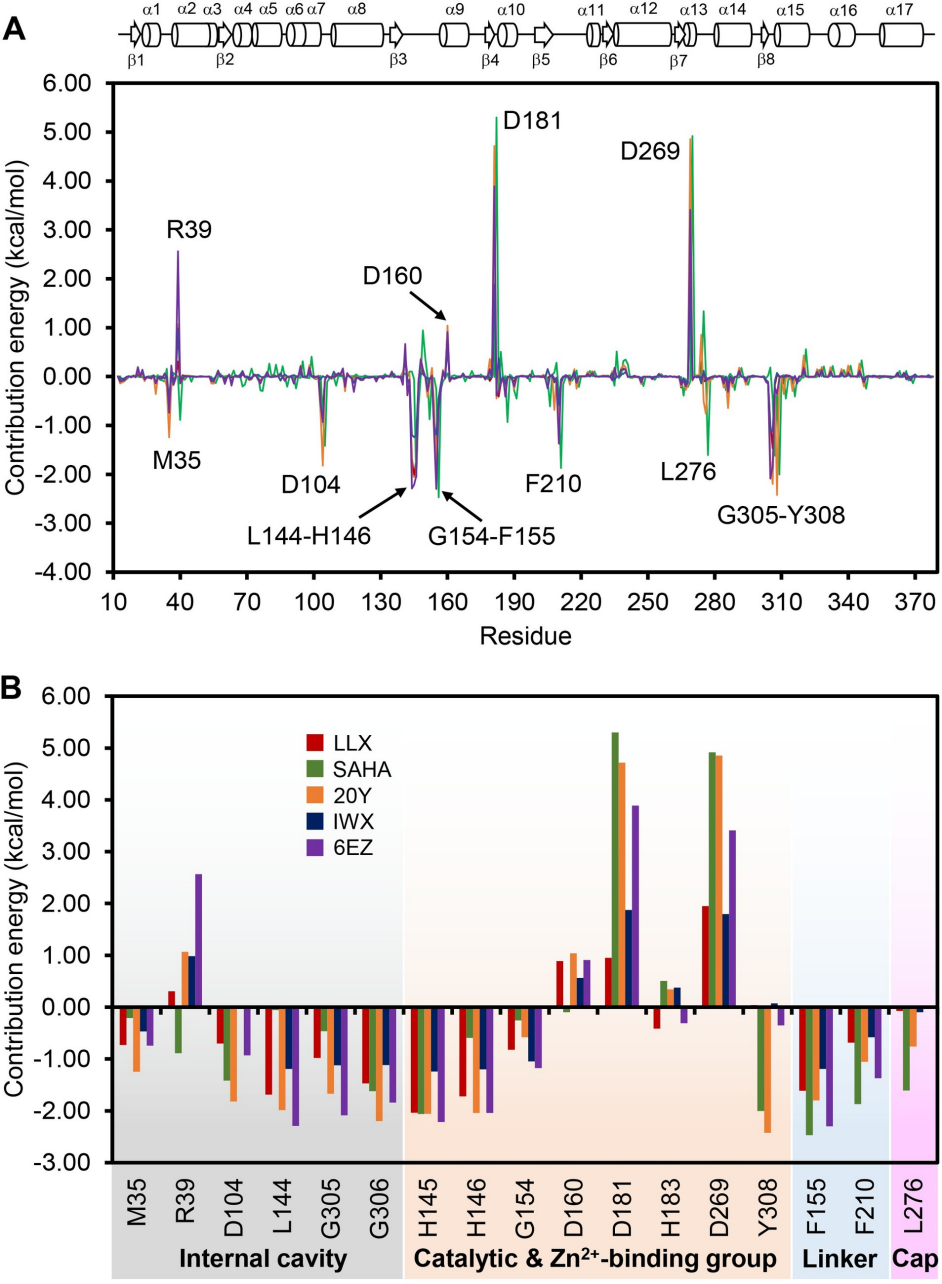
Decomposition of binding free energy per-residue of HDAC2 when bound by various inhibitors. (A) Contribution energies of all residues on HDAC2 are indicated when bound by either LLX (red), SAHA [60], 20Y (orange), IWX (blue), or 6EZ (purple) ligands. Topology diagram of HDAC2 is also depicted atop of the chart indicating the range of secondary structures along the residue sequence. Consensus of key residues that have significant contribution to the binding energy are identified by residue number and are specifically illustrated in more detail in a bar graph (B). Regions of the ligands those the key residues interact with are also indicated.

In order to pinpoint energetic contributions from each amino acid residue to the ligand binding in a more quantitative manner, we employed molecular mechanics Poisson-Boltzmann Surface Area (MM/PBSA) calculations to estimate the binding free energy per-residue. Fig 5A shows a decomposition plot of binding free energy contributed from individual binding residues of HDAC2 when bound by the five inhibitor ligands. It is apparent that most of the binding residues show favorable contribution energies (negative values) to the ligand binding as anticipated. However, a few charge-bearing residues within the active site give rise to unfavorable (positive values) contribution energies. These include the Zn^2+^-binding residues Asp181, His183, and Asp269, as well as the neighboring residues Asp160 and Arg39. Due to the fact that, these residues normally form a charge relay network with the cofactor in the absence of any ligand, it is possible that binding to a ligand that disrupts or interferes with this charge network could result in unfavorable energetic contributions. Interestingly, when looking at the trends of contribution energies from the two main Zn^2+^-binding residues Asp181 and Asp269 (Fig 5B), the least unfavorable values are found for the binding of LLX and IWX ligands, both of which are highly potent inhibitors with IC_50_ values of 27 nM and 62 nM, respectively. Of note, decomposition of non-bonding energies, namely electrostatic energy and van der Waals interaction, have been shown to correlate with the total binding energy of HDAC2 ligand binding [31], although without a consideration of entropic term. Our observation of the correlation between the energy decomposition and the actual potency thus confirms the role of per-residue energy as determinant factors for predicting the actual inhibitory activity as previously described [69–71].

Additionally, Fig 5B summarizes the contribution energies of amino acid residues found at sub-sites along the active-site cleft ranging from the internal foot pocket, catalytic residues (His145, His146, and Tyr308) and the Zn^2+^-binding group, the linker-binding, and the cap-binding regions. It is apparent that, in addition to the catalytic residues, several residues within the foot pocket, as well as the linker- and cap-binding sites, also contribute significantly to the ligand binding. Trends of contribution energies for each ligand correlate well with the binding mechanism outlined in the previous section describing intermolecular interactions. For example, binding of SAHA that primarily uses its aniline moiety to plug into the opening surface, but lacks the foot-pocket binding group, gives rise to the contribution energies that predominate in the linker- and cap-binding residues but less so in the internal cavity. On the other hand, binding of a potent ligand 20Y (IC_50_ = 56.3 nM) shows strong contribution energies in all regions. This coincides with the intermolecular interactions identified by a spatial visualization from the MD analysis (*vide supra*). Taken together, the results from this experiment suggest that the contribution energy per residue may be used in combination with the intermolecular analysis in order to explain a ligand binding mechanism and ultimately forecast its inhibitory potency. However, evaluation of additional thermodynamic parameters may also be required in order to construct a quantitative model to predict the inhibitory activity of a ligand family.

### Estimation of the entropic term

**Fig 6:**
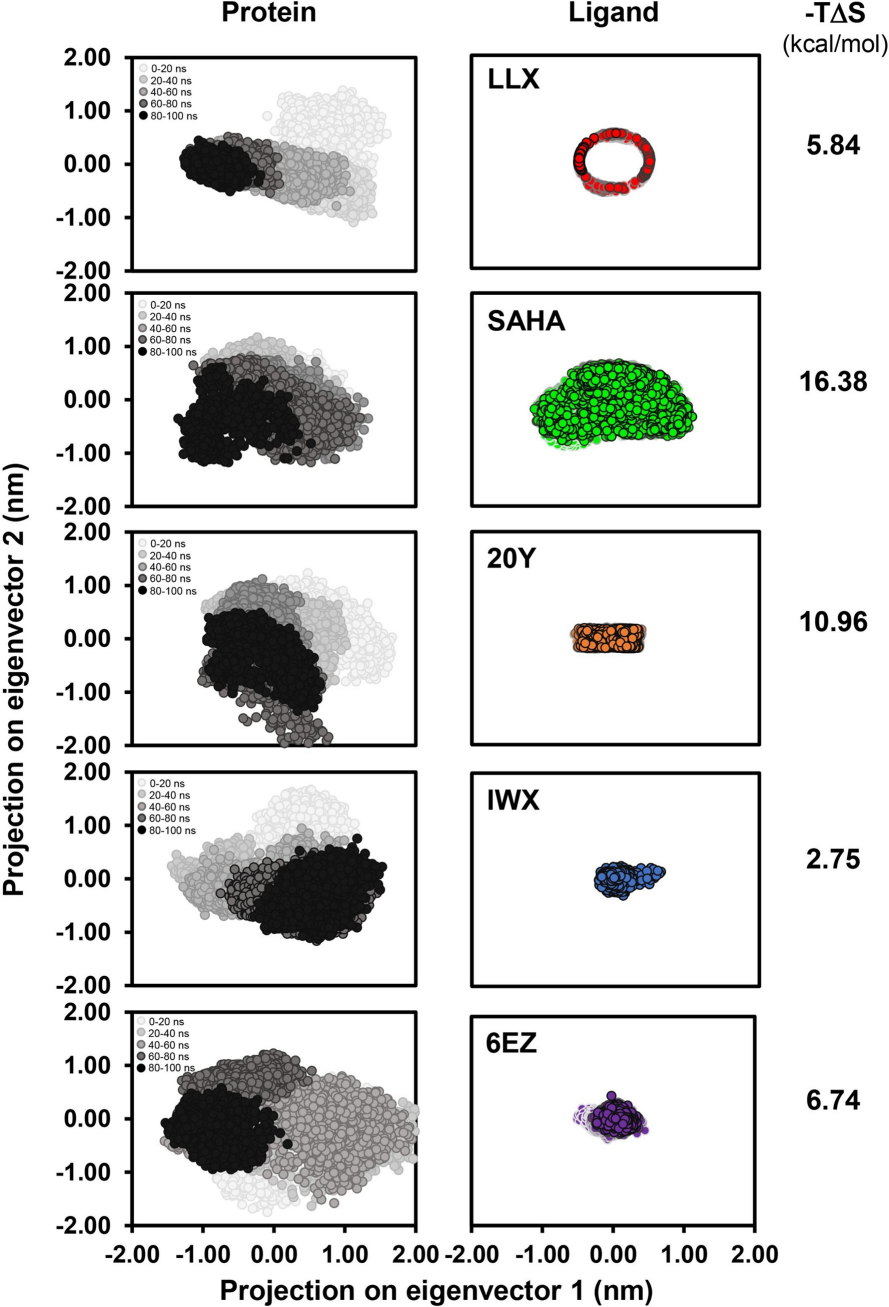
Two-dimensional projections of trajectory motions of the HDAC2 protein (left panels) and the bound ligands (right panels) in multidimensional space over the first two (PC1 and PC2) principal eigenvectors. Calculated -*T*Δ*S* values in kcal/mol of HDAC2-ligand complexes are shown on the right.

In order to extract additional information from the simulations, namely the entropic term of protein-ligand complex formation, the directions of molecular motions and fluctuations were first analyzed. The “essential dynamics method” was employed to examine conformational changes observed within the HDAC2 protein when bound by ligands of interest. PCA was performed via Gromacs inbuilt tool ‘g_covar’ in order to determine the concerted molecular motions, that resulted in a covariance matrix of backbone Cα atoms within the HDAC2-ligand complexes. Fig 6 shows two-dimensional projections of trajectory motions of the HDAC2 proteins when bound by the five ligands. Positive and negative limits of each dimension represent, respectively, correlated and anti-correlated motions of the backbone atoms in the same and opposite directions relative to the average positions. Subsequently, the covariance matrix was diagonalized to obtain the eigenvectors and eigenvalues, where the eigenvector with the highest eigenvalue is assigned as the first principal component, and the one with the second highest eigenvalue is considered the second principal component. The amplitude of eigenvectors along the spatial dimensions thus represents the conformational changes observed within the protein and the ligands.

The results show that conformational changes observed within the HDAC2 protein when bound by different ligands are similar in their amplitudes along the multidimensional spaces. However, HDAC2 when bound by 6EZ has slightly larger changes when compared to the others. On the other hand, when considering only ligand motions, SAHA was found to move around the most as the amplitudes on both directions are apparently larger than those found in other inhibitors. These observations could suggest that both HDAC2-6EZ and HDAC2-SAHA complexes may be less energetically stable. Interestingly, inhibitory activities of these two ligands are also the poorest among the five ligands, with IC_50_ values of 168 nM for 6EZ, and 251 nM for SAHA.

To further analyze the conformational results in a more quantitative manner, an entropic term was also derived and compared. To this end, an inbuilt Gromacs function ‘g_anaeig’ was employed to analyze the energy landscape for the two eigenvector projections and a quasi-harmonic approximation method was applied to derive the entropic term for each protein-ligand binding. The -*T*Δ*S* term was determined and displayed in the right column of Fig 6. It is apparent that the entropic term of all ligand complexes examined are rather different, with the highest being that of the HDAC2-SAHA complex. The vast difference in this term among the complexes piqued our curiosity: if the value of entropic term was added to the Δ*G*_binding_ equation (Eq 2), would improve the prediction of ligand binding and inhibitory activity?

### Comparison of the entropic contributions estimated among the different and similar ligand derivatives

**Fig 7:**
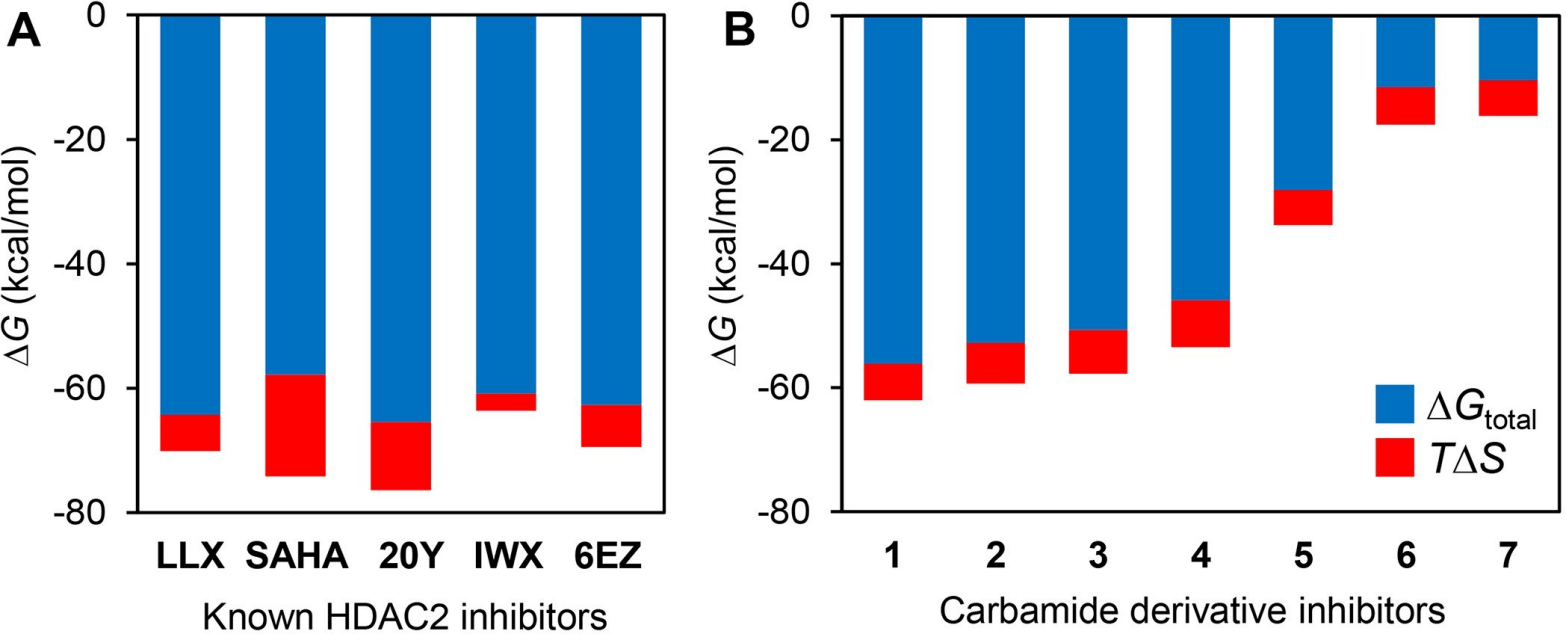
Binding free energies of either known inhibitors (A) or carbamide derivative inhibitors (B) comparing the scale of the entropic term contribution.

To help assess the level of energetic contribution from the entropic term to the overall binding free energy, additional energetic parameters were then collected. Aside from the binding free energy per-residue, the aforementioned MM/PBSA calculation also yielded other additional energetic terms that can be beneficial to our in-depth analysis. Table 1 summarizes the energetic parameters calculated from the MM/PBSA and the quasi-harmonic approximation of all HDAC2-inhbitor complexes. These parameters include *ΔE*_vdW_, *ΔE*_elec_, *ΔG*_polar_, *ΔG*_non-polar_, *ΔG*_PBSA_, and *-TΔS*, all of which amounted to the *ΔG*_total_ (Δ*G*_binding_). The related equations of each term are described in the Material and Methods section above. Fig 7A also compares the *ΔG*_total_ and the *TΔS* terms for all HDAC2-ligand complexes in a bar graph. When considering the sizes of both energetic terms determined in each complex, it is apparent that the entropic term contributes greatly to the total free energy of binding and including this term into the calculation could also change the trend of the *ΔG*_total_.

**Table 1:** Binding free energy of complexes between HDAC2 and known HDAC2 inhibitors based on 100-ns MD simulations and MM-PBSA calculations. The entropic contributions were calculated using the quasi-harmonic (QH) approximation. All values are presented in kcal/mol.

Energy	HDAC2-inhibitor complexes				
	LLX	SAHA	20Y	IWX	6EZ
***ΔE***_**vdW**_	-33.30	-26.27	-38.34	-39.34	-42.82
***ΔE***_**elec**_	-112.99	-103.15	-117.77	-101.20	-101.46
***ΔG***_**polar**_	80.06	58.62	84.07	80.91	79.20
***ΔG***_**non-polar**_	-3.86	-3.37	-4.37	-3.98	-4.36
***ΔG***_**PBSA**_	-70.08	-74.16	-76.42	-63.62	-69.44
***-TΔS***	5.84	16.32	10.96	2.75	6.74
***ΔG***_**total**_	-64.24	-57.84	-65.46	-60.87	-62.70

From the observation that the entropic term is rather important in a comparison of diverse ligands, we also wondered whether this pattern could also be seen among a set of structurally similar ligands. To this end, we performed the same set of experiments on seven additional carbamide derivative inhibitors of HDAC2 (chemical structures and energetic parameters shown in S4 Table of S1 File). These include molecular docking, MD simulations, MM/PBSA calculations, as well as the approximations of the entropic term. Table 2 summarizes the energetic parameters contributing to the total binding free energy of the complexes of carbamide scaffolds. Interestingly, the entropic terms found within the protein complexes for this structurally similar inhibitor molecules do not appear to be significantly different (Fig 7B). When accounting for both *ΔG*_total_ and *TΔS* terms of this set of ligands, it is obvious that addition of the entropic term does not change the trend of overall free energy of binding. Therefore, it is possible that, for deriving a trend among structurally similar ligands, including the entropic term may not be very critical. This possibility is being investigated in our laboratory with a larger sample size, and will be reported elsewhere.

**Table 2:** Binding free energy of complexes between HDAC2 and seven carbamate derivative inhibitors based on 100-ns MD simulations and MM-PBSA calculations. The entropic contributions were calculated using the quasi-harmonic (QH) approximation. All values are presented in kcal/mol.

Energy	Carbamate derivative compounds						
	1	2	3	4	5	6	7
***ΔE***_**vdW**_	-35.80	-34.62	-34.78	-33.53	-23.39	-21.77	-25.85
***ΔE***_**elec**_	-95.61	-88.20	-86.39	-90.26	-98.22	-97.43	-95.54
***ΔG***_**polar**_	73.21	66.92	66.96	70.51	90.89	104.25	108.19
***ΔG***_**non-polar**_	-3.8	-3.45	-3.54	-3.72	-3.03	-2.62	-2.98
***ΔG***_**PBSA**_	-62.00	-59.34	-57.75	-53.46	-33.75	-17.57	-16.17
***-TΔS***	5.88	6.57	7.08	7.60	5.64	6.07	5.75
***ΔG***_**total**_	-56.12	-52.77	-50.67	-45.86	-28.11	-11.49	-10.42

### Potential impact of the entropic term towards the inhibitory activity prediction

**Fig 8:**
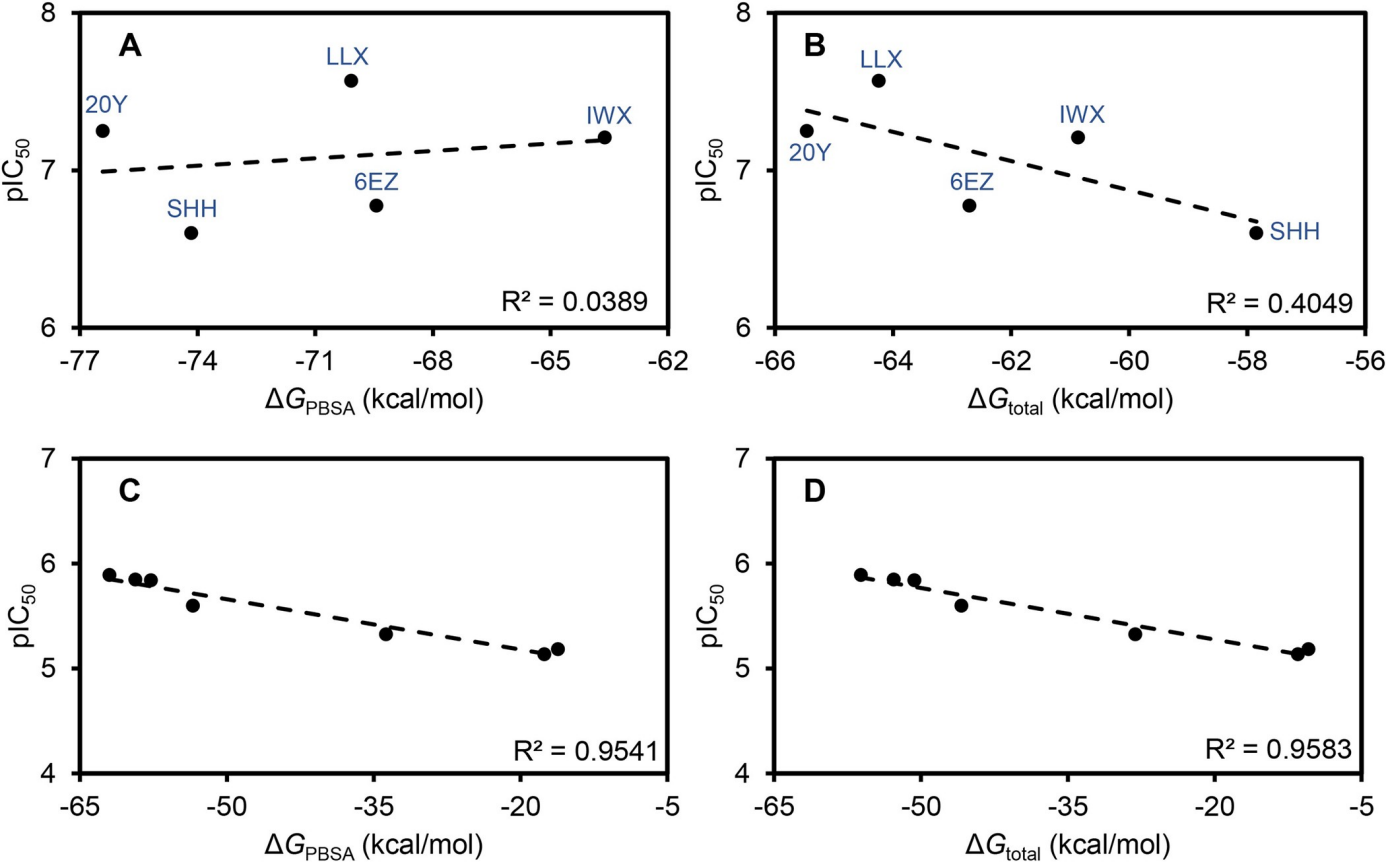
Correlation plots between experimental pIC_50_ values of HDAC2 inhibitors and the binding free energies of their complexes with HDAC2 calculated from MM-PBSA and additional entropic term estimations. Actual pIC_50_ potencies of known HDAC2 inhibitor ligands (A and B) or a set of carbamide derivatives (C and D) are plotted against the binding free energies of their complexes calculated from MM/PBSA alone (Δ*G*_PBSA_) (A and C) or calculated as total binding free energies by the addition of entropic term estimations (Δ*G*_total_) (B and D).

To further analyze the effect of entropic contribution, we then hypothesized that including the entropic term to calculate the Δ*G*_binding_ may alter a correlation plot between the binding free energy and the actual activity of the ligands. To test this hypothesis, we created a plot between the actual pIC_50_ values of HDAC2 inhibitors and their calculated binding free energy, with (Δ*G*_total_) or without the entropic term (Δ*G*_PBSA_) (Fig 8). Fig 8A and 8B compare the trends, respectively, before and after adding the values of -*TΔS* term. Although the data were analyzed with only these five different ligands, a trend can be observed when the entropic term was applied to the estimation of binding free energy. However, when considering the other set of structurally similar carbamide ligands before (Fig 8C) and after (Fig 8D) applying the entropic term, the correlation trends were not significantly different. It is also known in the literature that entropic contribution could be neglected when comparing a set of chemically related ligands, but not so for the unrelated ligands [72]. This agrees well with our previous observation that, for comparing derivative ligands, estimations of entropic terms may not be critical.

Additionally, it is noteworthy that the trend between experimental and computational data found within the similar ligands are already highly correlated. This could also imply a possibility that a prediction model created by energetic parameter calculation is more effective among similar ligands, but not so for diverse ligands. A larger pool of sample is needed, preferably from the same set of experiment, to fully examine this effect in the future.

Noticeably, the order of binding energies calculated from the ligand set changed slightly upon introducing the entropic term. Without the entropy, ΔG_PBSA_ energies ranks from low to high as 20Y < SHH < LLX < 6EZ < IWX, and with the entropy, the order of Δ*G*_total_ energies became 20Y < LLX < 6EZ < IWX < SHH. The change in the ranking of SHH was largely due to the consideration of entropic term, thus signifying the importance of the entropy calculation. However, as the experimental IC_50_ values range from low to high as LLX < 20Y < IWX < 6EZ < SHH, the calculated ranking does not correlate well with the experimental data. Additionally, the correlation remains relatively poor even when the entropic term was considered. This could suggest missing factors contributing to the accurate binding energy calculation.

Intriguingly, the correlation from the closely related ligands is relatively high even before the entropy was taken into account (Fig 8C and 8D), while the one from unrelated ligands remains poor. A potential explanation may include the fact that, for the unrelated ligands, MD simulations were performed based upon different crystal structures of HDAC2-ligand complexes, as we wanted to employ the most reliable structures available. This essentially differs from the MD simulations of related ligands as a single protein structure was used as a reference structure, and the ligands were docked into the same active site to generate initial structures for the MD simulations. Discrepancies in the protein structural coordinates among the crystal structure could contribute to the difference in the resulted MD trajectories and thus the calculation of entropic terms. This hypothesis will be tested further in our laboratory with a larger pool of related and unrelated ligands. A potential caveat may include the selection of a single crystal structure to be used as initial coordinates that could represent the entire population of legitimate crystal structures.

Another avenue for investigating the factors contributing to the total binding energy calculation is to consider solvation entropy in addition to the configurational entropy. Several studies have highlighted the role of solvation entropy in protein-ligand complex formation, namely the water molecules around the binding pockets [73–76]. However, a recent study also suggests that configurational entropy dominates over solvation entropy in the overall entropic contribution to ligand binding [77].

As aforementioned, the IC_50_ values reported from different laboratories, though some were from the same isozyme and using similar methods of determination, could varied greatly and thus resulted in uncorrelated trends (S8 Fig in S1 File). Future experiments could also benefit from using *K*_i_ values instead of IC_50_ as it is an absolute value that would not vary between experiments. However, for our study, *K*_i_ values could not be properly calculated using a theoretical conversion equation as the information of substrate concentration and the *K*_m_ values were not explicitly reported in most experiments we reviewed. Therefore, we used a set of reported IC_50_ values those determined by the most similar method of determination. Undoubtedly, for the purpose of prediction model construction in the future, a large number of *K*_i_ values, or IC_50_ values determined from the same experiment, should give rise to a more accurate prediction of inhibitory potency.

Employing molecular modeling to predict binding free energies currently helps accelerate drug development by lowering the time and effort required to generate a new set of candidate compounds. To date, there are several successful computational tools to calculate binding free energies in protein-ligand interactions. One of the state-of-the-art strategies is alchemical free energy methods [78–85] that showed a lot of promise in terms of facilitating the computation of binding free energies. Accurate relative free energies could identify whether ligand alterations have boosted affinity and selectivity, especially in lead optimization efforts. Generally, due to their higher rigor in evaluating interactions with explicit solvent and configurational entropy, alchemical approaches are recommended in later stages of drug optimization when fewer simulations of a restricted range of candidates are required [86]. On the other hand, MM/PBSA is recommended for the initial phases of virtual screening when dealing with a large number of chemical candidates. Although MM/PBSA provides less accurate results when compared with the alchemical methods, the technique requires less computational demand while providing higher accuracy than molecular docking, thus rendering it suitable for a preliminary estimation of binding free energies [87].

The modular nature of MM/PBSA and MM/GBSA, as well as the fact that these techniques do not require a training set in the calculations, make them appealing practices that have been successfully implemented to replicate and interpret experimental findings, as well as to improve virtual screening and docking results [87]. Nonetheless, the techniques contain several problematic approximations for estimating binding free energies, such as the lack of configurational entropy and data on the amount and free energy of water molecules in the binding site. Therefore, in our study, we further employed an estimation of the entropic term via the essential dynamics method in order to examine the concerted molecular motions, which can be translated to configuration entropy. Although this is not merely a method to obtain accurate values of both *ΔG*_total_ and *TΔS* terms, this simple technique could provide a trend of entropic contribution to the binding of a protein with structurally diverse or similar ligands, which could be beneficial to the initial screening of drug candidates. This is particularly critical for targeting proteins with multiple isoforms like the HDAC enzyme family because entropic contribution has been shown to be a selectivity driver for inhibitor binding [36]. It was found that the binding of several cycloalkenyl hydroxamates selective to HDAC6 exhibited significant entropic gain, whereas the binding of these compounds to HDAC8 showed substantial entropic loss, suggesting that entropy is a crucial contributor to the binding selectivity [36]. Hence, it is essential to consider the entropic term in the approximation of binding free energies even in an early stage of the compound screening.

## Conclusion

This study aimed to investigate the binding mechanism that the HDAC2 enzyme employs in differentiating various types of inhibitor ligands. MD simulation data provided a spatial visualization of how different ligands can interact with the sub-sites of the active site cleft. The level of energetic contribution per amino acid residues were estimated using MM/PBSA calculations, which resulted in a quantitative view of the binding behavior. It was found that the interactions within the internal foot pocket can contribute significantly to the ligand binding. Residues lining the opening rim of the active site also provide additional interactions to restrict the movement of ligand molecules. Protruding regions of the ligands lacking these contacts could cause a high fluctuation along the cap portion and thus weaken the overall ligand affinity. To further quantify this dynamics aspect, the entropic term was estimated and compared with the scale of Δ*G*_binding_. It was found that the values of -*T*Δ*S* term correlate with the level of disorder found within the ligand complexes. Furthermore, accounting for the entropic term in the estimation of Δ*G*_total_ could provide additional insight into its contribution to the binding energy. In particular, we found that the -*T*Δ*S* term may be necessary for a comparison of binding energy among a set of diverse ligands as the levels of their entropic contribution could greatly differ. However, for structurally similar ligands, this term appears within a comparable range and would unlikely change the ranking trend. This study has demonstrated the significance of molecular dynamics and an estimation of entropic contribution in describing inhibitor binding mechanism. Further development of the model to predict ligand affinities and inhibitory activity could thus be pursued in order to help guiding an optimization of ligand candidates to be developed in future drug discovery campaigns.

## Supporting information

S1 File S1 Fig. Root-mean-square deviations (RMSD) of backbone atoms on HDAC2 when bound or unbound with inhibitors, comparing between two independent MD repeats. Unbound HDAC2 (A) or HDAC2 in complex with LLX (B), SAHA (C), 20Y (D), IWX (E), and 6EZ (F) are separately illustrated. S2 Fig. Root-mean-square deviations (RMSD) of backbone atoms on HDAC2 when bound with 20Y ligand, comparing the ranges of fluctuation during the original 100-ns MD run, as used in this study, and the extended 200-ns MD run. S3 Fig. Overlay of snapshot structures of HDAC2 when bound with 20Y ligand obtained at 100 ns and at 200 ns of the MD simulation. RMSD between the heavy atoms of the two structure is 0.8187 Å. S4 Fig. Binding interactions between inhibitor ligands and the active site of HDAC2, highlighting metal chelation with the Zn^2+^ cofactors. Inhibitor ligands LLX (A), SAHA (B), 20Y (C), IWX (D), and 6EZ (E) are shown in stick representations. Zn^2+^ cofactors are shown as purple spheres. S5 Fig. The distribution of distances between the Zn^2+^ cofactor of HDAC2 and a nearby heavy atom of amino acid residues or ligands. S6 Fig. Radius distribution function (RDF), detected during the 100-ns simulations, for the Zn^2+^ ion as a result of interactions with its atomic partners within the catalytic site of HDAC2, which include Asp181-OD1 (blue), Asp181-OD2 [88], His183-ND1 (orange), Asp269-OD1 [60], Asp269-OD2 (brown), and the bound inhibitors (blue and red). S7 Fig. Running coordination number, detected during the 100-ns simulations, for the Zn^2+^ ion as a result of interactions with its atomic partners within the catalytic site of HDAC2, which include Asp181-OD1 (blue), Asp181-OD2 [88], His183-ND1 (orange), Asp269-OD1 [60], Asp269-OD2 (brown), and the bound inhibitors (blue and red). S8 Fig. Correlation plots between experimental pIC_50_ values of HDAC2 inhibitors, collected from previous studies indicated in S5 Table, and the binding free energies of their complexes with HDAC2 calculated from MM-PBSA and additional entropic term estimations. Actual pIC_50_ potencies of known HDAC2 inhibitor ligands are plotted against the binding free energies of their complexes calculated from MM/PBSA alone (Δ*G*_PBSA_)(A) or calculated as total binding free energies by the addition of entropic term estimations (Δ*G*_total_)(B). Grey dots represent the pIC_50_ values reported in the various studies, while black dots represent the average value for each inhibitor. S1 Table Protonation states of histidine residue of HDAC2 in pH 7.0 as calculated from the PropKa server (https://www.ddl.unimi.it/vegaol/propka.htm). A histidine can adopt three protonation states: HIP (+1 charged, both δ- and ε-nitrogens protonated), HID (neutral, δ-nitrogen protonated), and HIE (neutral, ε-nitrogen protonated). S2 Table Atomic distances between the Zn^2+^ cofactor of HDAC2 and a nearby heavy atom of amino acid residues or ligands. Values are shown as average distance ± standard deviation. S3 Table Comparison between biochemical activity (IC_50_) and *in silico* information of known HDAC2 inhibitors. S4 Table Comparison between biochemical activity (IC_50_)^a^ and *in silico* information of HDAC2 carbamide derivative inhibitors. S5 Table Comparison of reported IC_50_ values of HDAC inhibitors and the experimental methods used in the determination of inhibitory potency. Values labeled with an asterisk are the values selected for the use in our calculation, which are results from the most similar methods of determination.
(PDF)
