# Supplementary material for: Decoding molecular recognition of inhibitors targeting HDAC2 via molecular dynamics simulations and configurational entropy estimation
Source: PLoS One. 2022 Aug 18;17(8):e0273265. doi: 10.1371/journal.pone.0273265 (PMC9387782; doi:10.1371/journal.pone.0273265)
Supplement: S1 File — S1 Fig. Root-mean-square deviations (RMSD) of backbone atoms on HDAC2 when bound or unbound with inhibitors, comparing between two independent MD repeats. Unbound HDAC2 (A) or HDAC2 in complex with LLX (B), SAHA (C), 20Y (D), IWX (E), and 6EZ (F) are separately illustrated. S2 Fig. Root-mean-square deviations (RMSD) of backbone atoms on HDAC2 when bound with 20Y ligand, comparing the ranges of fluctuation during the original 100-ns MD run, as used in this study, and the extended 200-ns MD run. S3 Fig. Overlay of snapshot structures of HDAC2 when bound with 20Y ligand obtained at 100 ns and at 200 ns of the MD simulation. RMSD between the heavy atoms of the two structure is 0.8187 Å. S4 Fig. Binding interactions between inhibitor ligands and the active site of HDAC2, highlighting metal chelation with the Zn2+ cofactors. Inhibitor ligands LLX (A), SAHA (B), 20Y (C), IWX (D), and 6EZ (E) are shown in stick representations. Zn2+ cofactors are shown as purple spheres. S5 Fig. The distribution of distances between the Zn2+ cofactor of HDAC2 and a nearby heavy atom of amino acid residues or ligands. S6 Fig. Radius distribution function (RDF), detected during the 100-ns simulations, for the Zn2+ ion as a result of interactions with its atomic partners within the catalytic site of HDAC2, which include Asp181-OD1 (blue), Asp181-OD2 [88], His183-ND1 (orange), Asp269-OD1 [60], Asp269-OD2 (brown), and the bound inhibitors (blue and red). S7 Fig. Running coordination number, detected during the 100-ns simulations, for the Zn2+ ion as a result of interactions with its atomic partners within the catalytic site of HDAC2, which include Asp181-OD1 (blue), Asp181-OD2 [88], His183-ND1 (orange), Asp269-OD1 [60], Asp269-OD2 (brown), and the bound inhibitors (blue and red). S8 Fig. Correlation plots between experimental pIC50 values of HDAC2 inhibitors, collected from previous studies indicated in S5 Table, and the binding free energies of their complexes with HDAC2 calculated from MM-PBSA [file pone.0273265.s001.pdf]

Supporting information

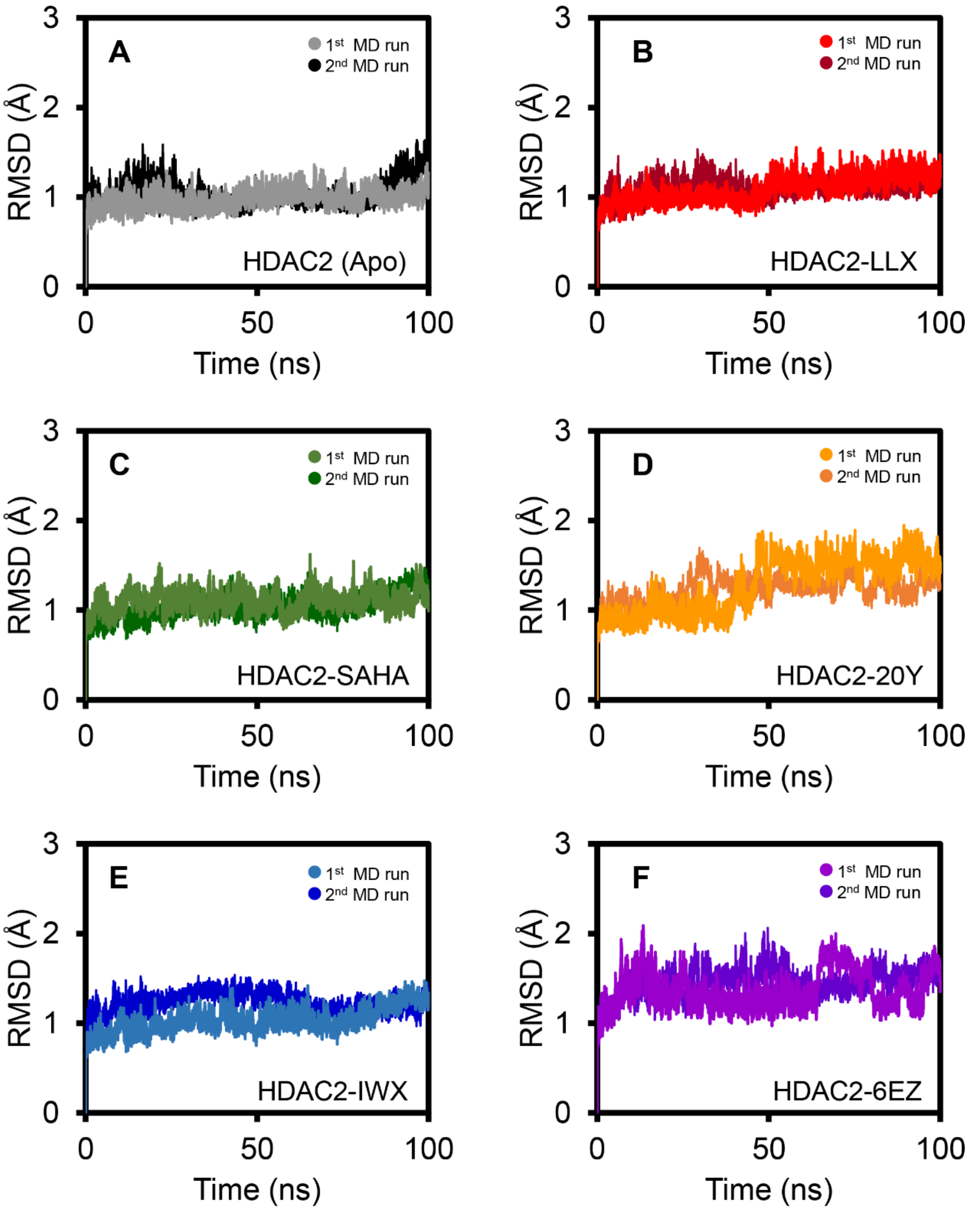

S1 Fig. Root-mean-square deviations (RMSD) of backbone atoms on HDAC2 when bound or unbound with inhibitors, comparing between two independent MD repeats. Unbound HDAC2 (**A**) or HDAC2 in complex with LLX (**B**), SAHA (**C**), 20Y (**D**), IWX (**E**), and 6EZ (**F**) are separately illustrated.

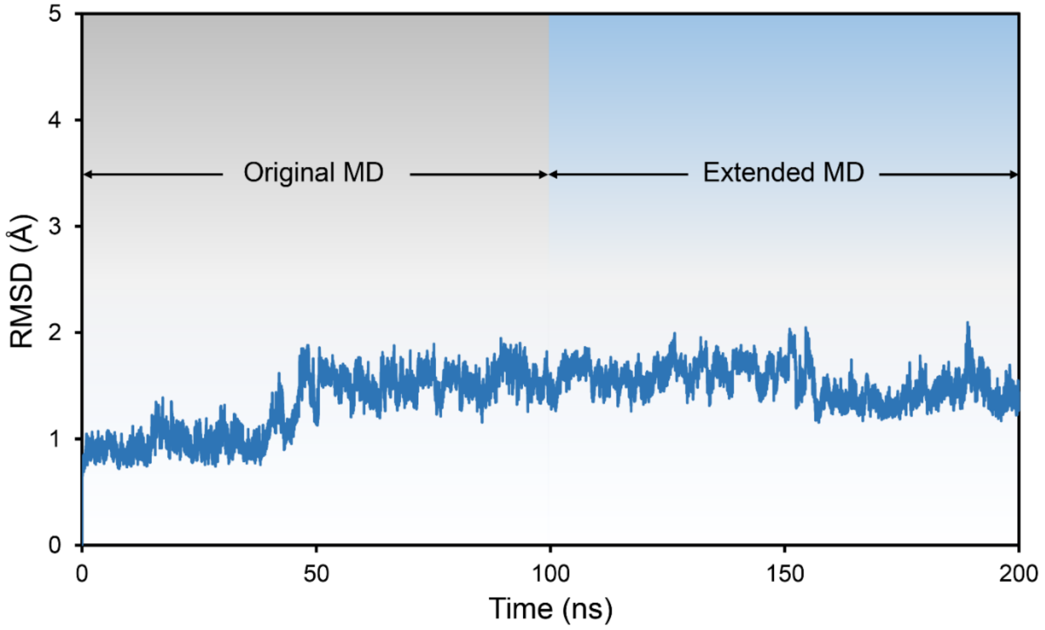

S2 Fig. Root-mean-square deviations (RMSD) of backbone atoms on HDAC2 when bound with 20Y ligand, comparing the ranges of fluctuation during the original 100-ns MD run, as used in this study, and the extended 200-ns MD run.

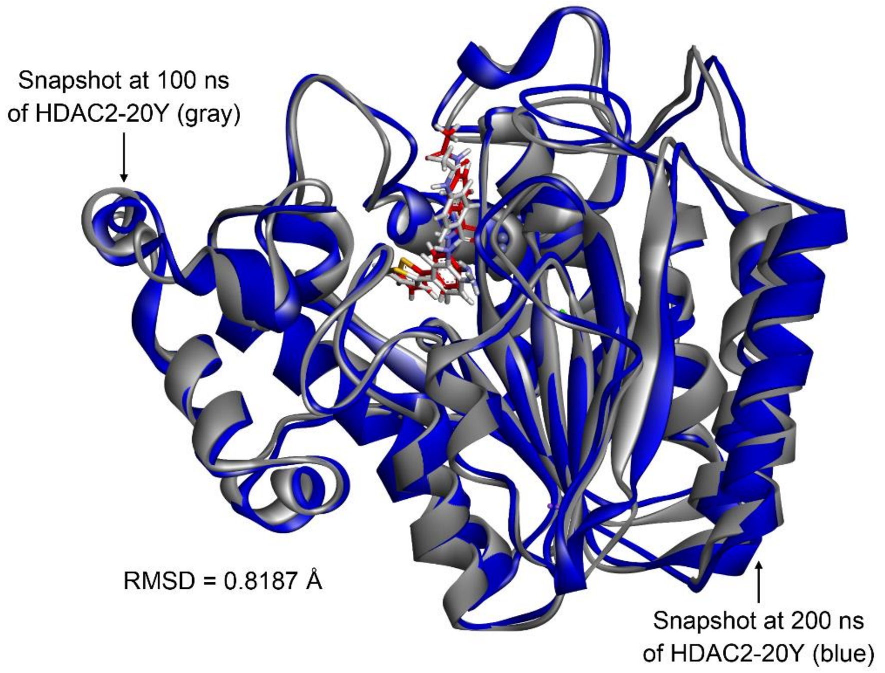

S3 Fig. Overlay of snapshot structures of HDAC2 when bound with 20Y ligand obtained at 100 ns and at 200 ns of the MD simulation. RMSD between the heavy atoms of the two structure is 0.8187 Å.

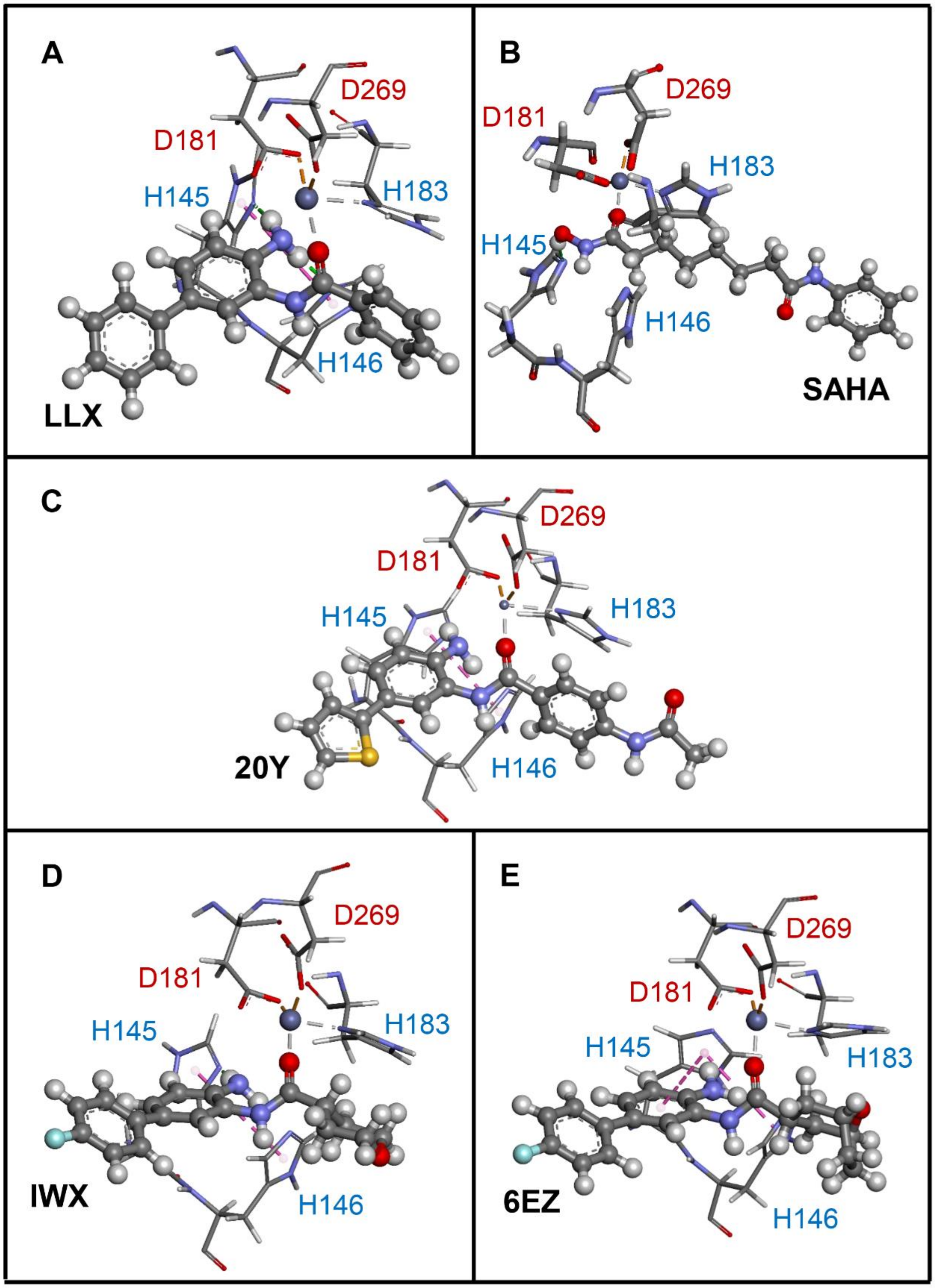

S4 Fig. Binding interactions between inhibitor ligands and the active site of HDAC2, highlighting metal chelation with the Zn^{2+} cofactors. Inhibitor ligands LLX (A), SAHA (B), 20Y (C), IWX (D), and 6EZ (E) are shown in stick representations. Zn^{2+} cofactors are shown as purple spheres.

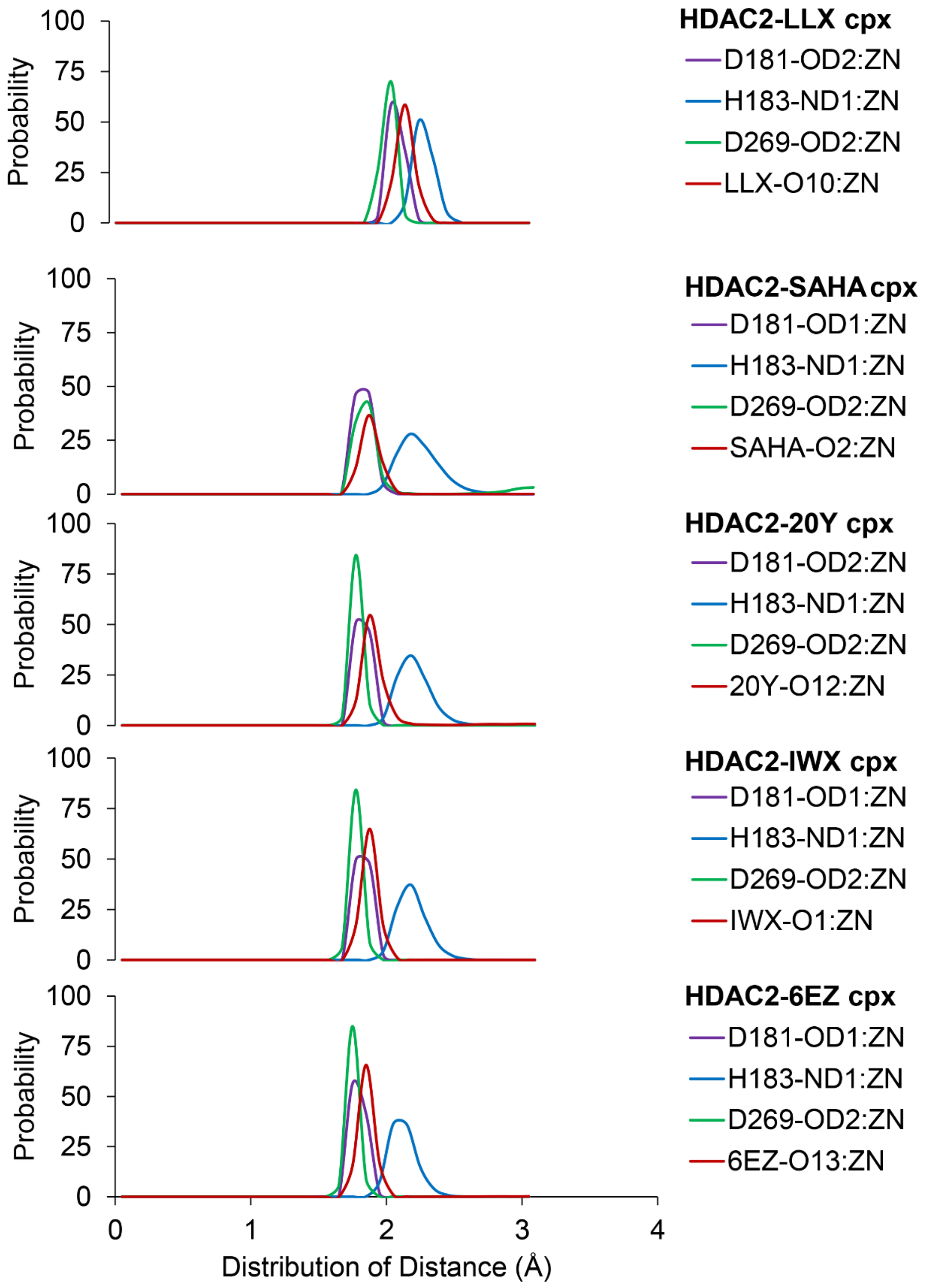

S5 Fig. The distribution of distances between the Zn^{2+} cofactor of HDAC2 and a nearby heavy atom of amino acid residues or ligands.

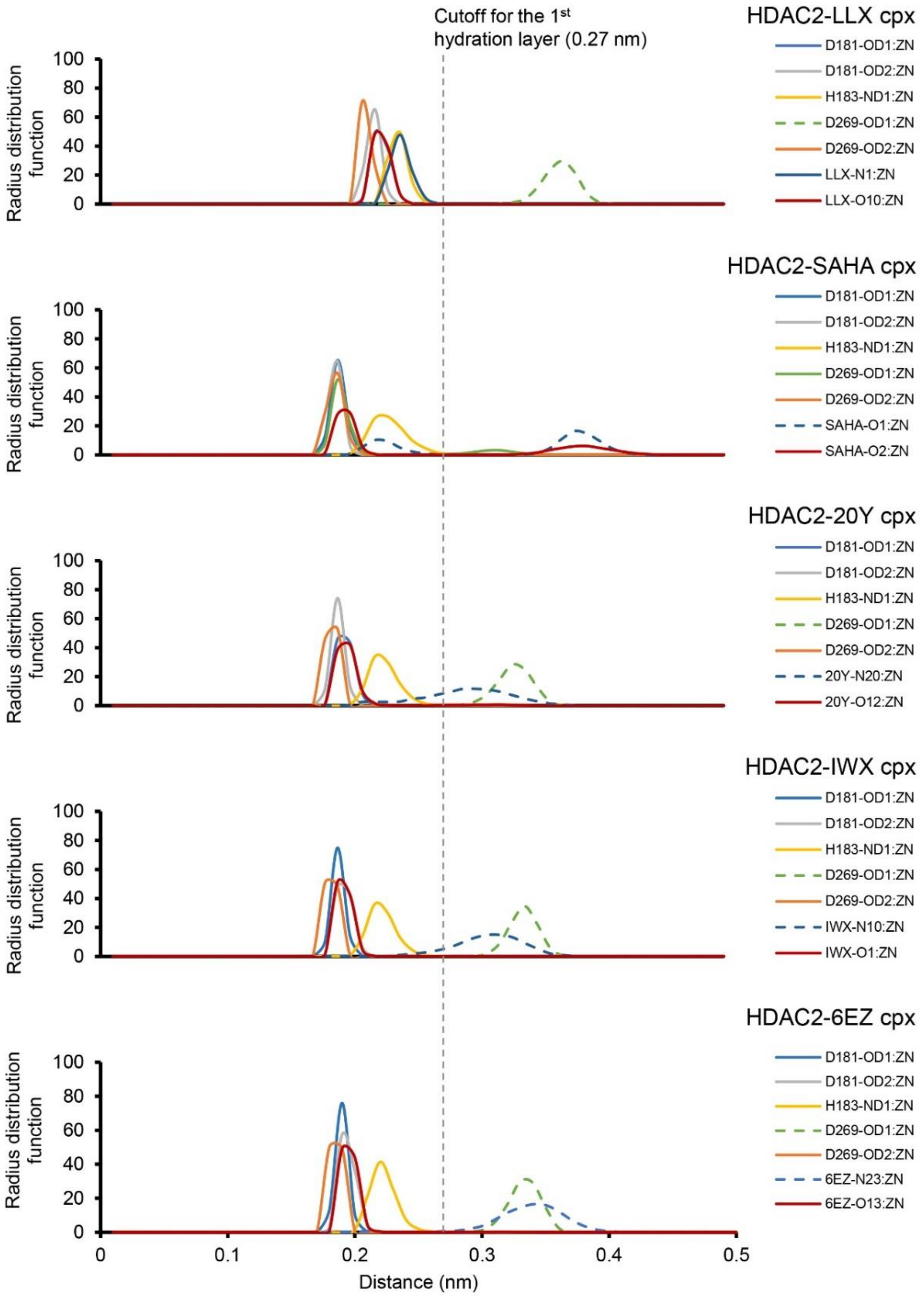

S6 Fig. Radius distribution function (RDF), detected during the 100-ns simulations, for the Zn^{2+} ion as a result of interactions with its atomic partners within the catalytic site of HDAC2, which include Asp181-OD1 (blue), Asp181-OD2 (gray), His183-ND1 (orange), Asp269-OD1 (green), Asp269-OD2 (brown), and the bound inhibitors (blue and red).

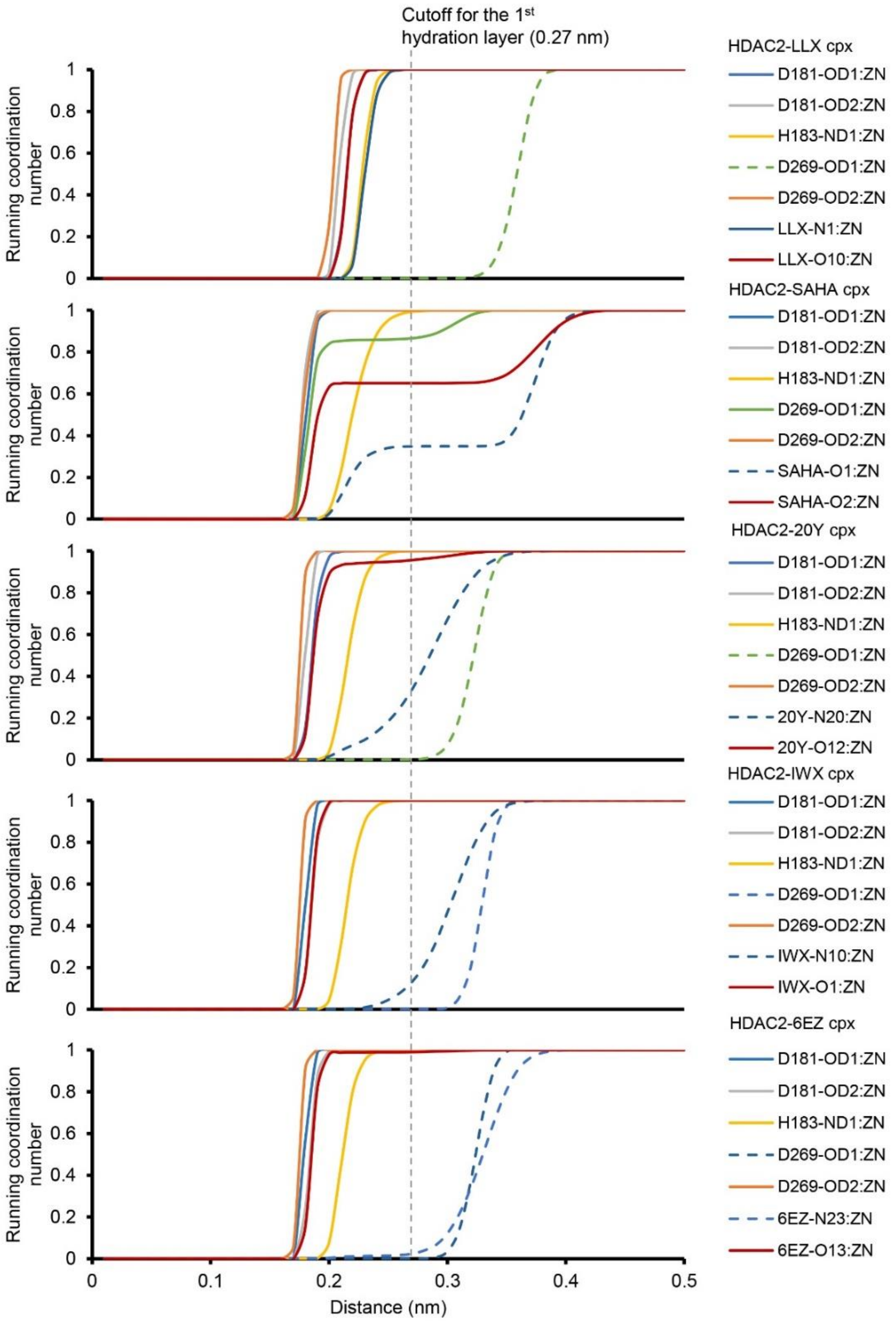

S7 Fig. Running coordination number, detected during the 100-ns simulations, for the Zn^{2+} ion as a result of interactions with its atomic partners within the catalytic site of HDAC2, which include Asp181-OD1 (blue), Asp181-OD2 (gray), His183-ND1 (orange), Asp269-OD1 (green), Asp269-OD2 (brown), and the bound inhibitors (blue and red).

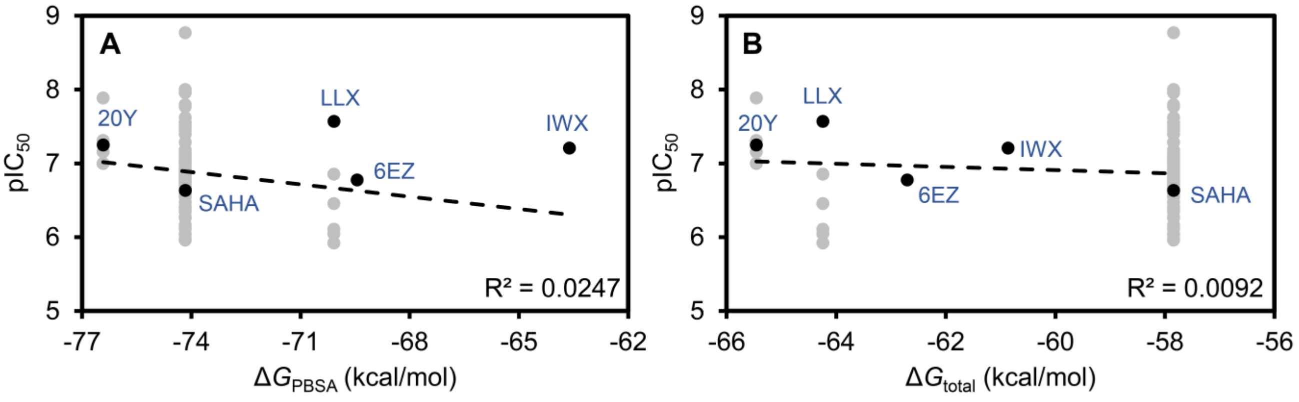

S8 Fig. Correlation plots between experimental pIC_{50} values of HDAC2 inhibitors, collected from previous studies indicated in table S5, and the binding free energies of their complexes with HDAC2 calculated from MM-PBSA and additional entropic term estimations. Actual pIC_{50} potencies of known HDAC2 inhibitor ligands are plotted against the binding free energies of their complexes calculated from MM/PBSA alone (ΔG_{PBSA})(**A**) or calculated as total binding free energies by the addition of entropic term estimations (ΔG_{total})(**B**). Grey dots represent the pIC_{50} values reported in the various studies, while black dots represent the average value for each inhibitor.

S1 Table Protonation states of histidine residue of HDAC2 in pH 7.0 as calculated from the PropKa server (https://www.ddl.unimi.it/vegaol/propka.htm). A histidine can adopt three protonation states: HIP (+1 charged, both δ- and ε-nitrogens protonated), HID (neutral, δ-nitrogen protonated), and HIE (neutral, ε-nitrogen protonated).

Histidine residue	pK_a value	Histidine Protonation State
His33	5.35	HIE
His38	4.67	HIE
His44	4.15	HID
His62	6.88	HIE
His73	3.53	HID
His145	6.84	HID
His146	1.50	HID
His172	5.14	HID
His183	4.18	HID
His184	6.23	HIE
His204	0.15	HIE
His286	7.80	HIP
His349	4.46	HID

S2 Table Atomic distances between the Zn^{2+} cofactor of HDAC2 and a nearby heavy atom of amino acid residues or ligands. Values are shown as average distance ± standard deviation.

	Paired atoms	Metal chelation (Å)				
		**3MAX** (HDAC-LLX)	**4LXZ** (HDAC-SAHA)	**4LY1** (HDAC-20Y)	**5IWG** (HDAC-IWX)	**5IW0** (HDAC-6EZ)
**HDAC2 enzyme**	D181-OD1 : Zn^{2+}	-	1.81±0.05	-	1.80±0.04	1.80±0.04
	D181-OD2 : Zn^{2+}	2.09±0.05	-	1.80±0.04	±	±
	H183-ND1 : Zn^{2+}	2.29±0.07	2.22±0.15	2.17±0.12	2.16±0.01	2.12±0.10
	D269-OD2 : Zn^{2+}	2.03±0.04	1.78±0.06	1.76±0.03	1.75±0.03	1.75±0.03
**HDAC2 inhibitor**	LLX-O10 : Zn^{2+}	2.15±0.06	-	-	-	-
	SAHA-O2 : Zn^{2+}	-	2.52±0.92	-	-	-
	20Y-O12 : Zn^{2+}	-	-	1.93±0.26	-	-
	IWX-O1 : Zn^{2+}	-	-	-	1.85±0.05	-
	6EZ-O13 : Zn^{2+}	-	-	-	-	1.87±0.14

S3 Table Comparison between biochemical activity (IC_{50}) and *in silico* information of known HDAC2 inhibitors

Cpd.	2D structure	IC_{50} (nM)	ΔG_{PBSA} (kcal/mol)	$-T\Delta S$ (kcal/mol)	ΔG_{total} (kcal/mol)
**LLX**	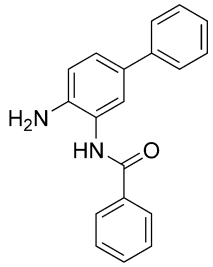	27[a]	-70.08 ± 2.24	5.84	-63.24 ± 2.24
**SAHA**	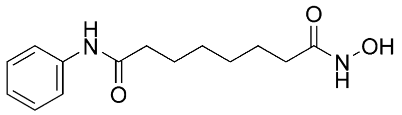	251[b]	-74.16 ± 3.69	16.32	-57.84 ± 3.69
**20Y**	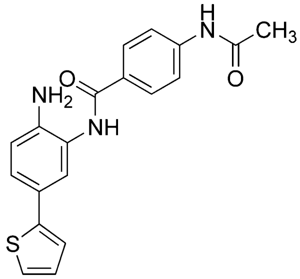	56.3[b]	-76.42 ± 5.42	10.96	-65.46 ± 5.42
**IWX (BRD4884)**	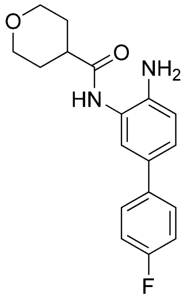	62 ± 31[c]	-63.62 ± 4.54	2.75	-60.87 ± 4.54
**6EZ (BRD7232)**	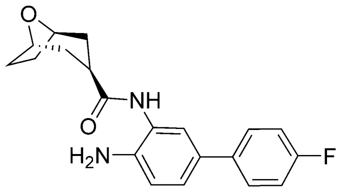	168 ± 52[c]	-69.44 ± 6.05	6.74	-62.70 ± 6.05

[a] Data from ref. [1]
[b] Data from ref. [2]
[c] Data from ref. [3]

S4 Table Comparison between biochemical activity (IC_{50})[a] and *in silico* information of HDAC2 carbamide derivative inhibitors

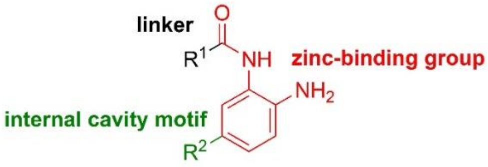

Cpd.	2D structure	IC_{50}[c] (μM)	ΔG_{PBSA} (kcal/mol)	$-T\Delta S$ (kcal/mol)	ΔG_{total} (kcal/mol)
**1**	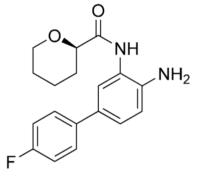	1.29 ± 0.399	-62.00 ± 5.10	5.88	-56.12 ± 5.10
**2**	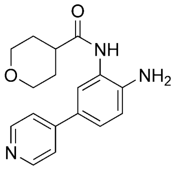	1.42 ± 0.443	-59.34 ± 4.84	6.57	-52.77 ± 4.84
**3**	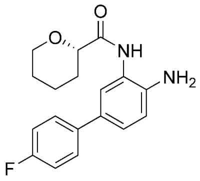	1.45 ± 0.486	-57.75 ± 5.22	7.08	-50.67 ± 5.22
**4**	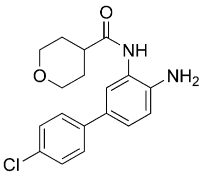	2.53 ± 0.661	-53.46 ± 5.46	7.60	-45.86 ± 5.46
**5**	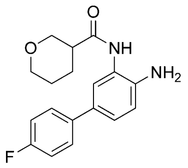	4.73 ± 0.299	-33.75 ± 5.88	5.64	-28.11 ± 5.10
**6**	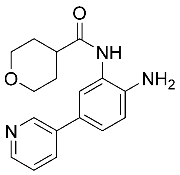	7.32 ± 1.04	-17.57 ± 5.16	6.07	-11.49 ± 5.22
**7**	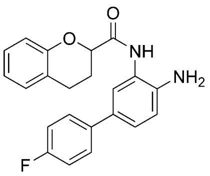	6.56 ± 2.66	-16.17 ± 5.18	5.75	-10.42 ± 5.16

[a] Data from ref. [3]

S5 Table Comparison of reported IC_{50} values of HDAC inhibitors and the experimental methods used in the determination of inhibitory potency. Values labeled with an asterisk are the values selected for the use in our calculation, which are results from the most similar methods of determination.

HDAC2-inhibitors	IC_{50} (nM)	Methods of Determination	Reference
LLX (6 assays)	27*	Recombinant HDAC2 assay (preincubated for 24 hrs, reaction 1 hrs)	[1]
	140	Recombinant HDAC2 assay (preincubated for 24 hrs, reaction 35 mins)	[4]
	350	Recombinant HDAC2 assay (preincubated for 3 hrs, reaction 35 mins)	[4]
	780	Inhibition of HDAC2	[5]
	900	Recombinant HDAC2 assay (preincubated for 1 hrs, reaction 1 hrs)	[1]
	1,200	Recombinant HDAC2 assay (preincubated for 5 mins, reaction 35 mins)	[4]
SAHA (114 assays)	1.7	Inhibition of human recombinant HDAC2 after 30 mins by fluorometric assay	[6]
	10	Inhibition of human recombinant HDAC2 using acetyllysine tripeptide coupled with 7-amino-4-methylcoumarin as substrate by fluorescence assay	[7]
	10	Inhibition of human histone deacetylase (mixture of HDAC1 and HDAC2) prepared from K562 erythroleukemia cells.	[8]
	11	Purified HDAC1-9 (0.5~5 nM) were incubated with 2 μM carboxyfluorescein (FAM)-labeled acetylated peptide substrate A or B	[9]
	11	All histone deacetylases were purchased from BPS Bioscience. The substrates, Broad Substrate A, and Broad Substrate B	US10188756 (2019)
	11	Inhibition of HDAC2 (unknown origin) assessed as fluorescence intensity measured after 60 mins incubation at room temperature	[10]
	11	Inhibition of HDAC1/HDAC2 in human HeLa cell nuclear extract preincubated for 20 mins followed by addition of HDAC green as substrate	[11]
	16	Inhibition of human recombinant full-length HDAC2 using fluorophore conjugated substrate by fluorescence assay	[12]
	17	Inhibition of HDAC2 (unknown origin) using Ac-Leu-GlyLys(Ac)-AMC as substrate preincubated for 10 mins followed by substrate addition	[13]
	24	Inhibition of human recombinant HDAC2 using fluor de Lys as substrate by fluorometric analysis	[14]
	28	Inhibition of human HDAC2 pre-incubated for 30 mins before substrate addition and measured after 30 mins by HDAC-Glo I/II assay	[15]

HDAC2-inhibitors	IC_{50} (nM)	Methods of Determination	Reference
	28	Inhibition of C-terminal His-tagged full length human HDAC2 expressed in baculovirus expression system using Ac-peptide substrate incubated for 15 mins	[16]
	31	Inhibition of full length recombinant human C-terminal His-tagged HDAC2 expressed in baculovirus infected sf9 cells using Boc-Lys-(Ac)-AMC as substrate	[17]
	32	Inhibition of human recombinant HDAC2 using Ac-Leu-Gly-Lys(Ac)-AMC as substrate preincubated for 30 mins followed by substrate addition	[18]
	35	Inhibition of recombinant HDAC2 (unknown origin) using AMC labeled AC-peptide as substrate incubated for 1 hr by fluorescence analysis	[19]
	36	Inhibition of HDAC2	[20]
	40	Inhibition of HDAC2 (unknown origin)	[21]
	52	Inhibition of human recombinant HDAC2 using MAZ1600 as fluorogenic substrate measured every 5 mins by optimized homogenous fluorescence-based assay	[22]
	66	Inhibition of full length HDAC2 assessed as 7-amino-4-methylcoumarin release from fluorophore conjugated substrate after 5 mins by fluorescence assay	[23]
	66	Inhibition of recombinant human HDAC2 using Fluor-de-Lys as substrate after 30 mins by spectrophotometry	[24]
	72	Inhibition of human recombinant HDAC2 preincubated for 24 hrs followed by 1 hr reaction with substrate by protease coupled end-point assay	[1]
	73	Inhibition of Histone deacetylase 2 (HDAC2) activity of HeLa nuclear extracts	[25]
	76	Inhibition of human recombinant HDAC2 preincubated for 1 hr with substrate by protease coupled end-point assay	[1]
	81	Inhibition of VLA-4 from HL60 lysate in a protein-based ligand binding assay	[26]
	81	Inhibition of C-terminal GST-tagged human recombinant HDAC2 expressed in Sf9 cells after 30 mins by microtiter plate reader analysis	[27]
	82	The Fluor-de-Lys HDAC activity assay kit (Biomol) was used. Purified recombinant HDAC enzyme was incubated with Fluor-de-Lys substrate	[28]
	82	Inhibition of C-terminal FLAG tagged HDAC2 (unknown origin)	[29]
	82	Inhibition of human C-terminal FLAG-tagged HDAC2 in HEK293 cells	[30]
	82	Inhibition of HDAC2	[31]
	86	Inhibition of human recombinant HDAC2 after 30 mins by fluorescence assay	[32]
	91	Inhibition of HDAC1/2 in human HeLa cell nuclear extract using color de Lys as substrate preincubated for 5 mins followed by substrate addition	[27]

HDAC2-inhibitors	IC_{50} (nM)	Methods of Determination	Reference
	92	Inhibition of human recombinant full-length C-terminal GST-tagged HDAC2 expressed in baculovirus infected Sf9 cells using Boc-Lys(acetyl)-AMC as substrate	[33]
	96	Inhibition of HDAC2 in human HeLa-S3 cell lysates preincubated for 15 mins followed by HDAC-Glo substrate addition measured after 30 to 40 mins by fluorescence assay	[34]
	96	Inhibition of HDAC2 (unknown origin) by ELISA-based assay	[35]
	96	Inhibition of HDAC2 in human HeLaS3 cells preincubated for 15 mins followed by HDAC-Glo substrate addition measured after 30 to 45 mins by ELISA	[35]
	100	Inhibition of human recombinant HDAC2 using fluorophore-conjugated substrate Boc-L-Lys(Ac)-AMC after 60 mins	[36]
	100	Inhibition of HDAC2 in human HeLa cells	[37]
	100	Inhibition of human recombinant HDAC2 using Fluor de Lys Green as substrate incubated for 30 mins by fluorescence assay	[38]
	100	Inhibition of HDAC2 in human HeLa cells using KI-104 as substrate after 40 mins by fluorescence analysis	[39]
	101	The HDAC activity was performed using the HDAC fluorescent activity assay kit (BIOMOL, Plymouth Meeting, Pa., USA) according to the manufacture	US9353061 (2016)
	101	The HDAC activity was performed using the HDAC fluorescent activity assay kit (BIOMOL, Plymouth Meeting, Pa., USA) according to the manufacturer's instruction	US9115116 (2015)
	101	Inhibition of recombinant HDAC2 (unknown origin) after 10 mins by fluorimetric analysis	[40]
	108	Inhibition of HDAC1/2 in human HeLa cells using Boc-Lys (acetyl)-AMC as substrate preincubated for 30 mins followed by substrate addition	[41]
	118	Inhibition of HDAC2 (unknown origin)	[42]
	120	Inhibition of recombinant human full length HDAC2 using Fluor-de-Lys as substrate after 60 mins by spectrofluorimetric analysis	[43]
	120	Inhibition of full length recombinant human HDAC2 expressed in baculovirus expression system assessed as release of 7-amino-4-methylcoumarin by fluorimetric analysis	[44]
	120	Inhibition of HDAC1/HDAC2 in human HeLa cells nuclear extract preincubated for 5 mins before Boc-Lys (acetyl)-AMC substrate addition for 30 mins	[45]
	121	Inhibition of full length human recombinant HDAC2 expressed in baculovirus infected insect S9 cells using Ac-Leu-GlyLys(Ac)-AMC as substrate addition	[46]
	130	Inhibition of HDAC1/2 in human HeLa cell extracts using acetylated histone peptide as substrate after 30 mins	[47]

HDAC2-inhibitors	IC_{50} (nM)	Methods of Determination	Reference
	135	Inhibition of recombinant human HDAC2 using Boc-Lys(Ac)-AMC as substrate preincubated for 15 mins followed by substrate addition measured after 60 mins	[48]
	138	Inhibition of HDAC1/2 in human HeLa nuclear extract using Boc-Lys(acetyl)-AMC as substrate preincubated for 5 mins followed by substrate addition	[49]
	140	Inhibition of HDAC2 (unknown origin)	[50]
	140	Inhibitory activity against histone deacetylase (HDAC1 and HDAC2) isolated from K562 erythroleukemia cells	[51]
	143	Inhibitory activity was tested against histone deacetylase (HDAC)	[52]
	149	Inhibition of recombinant HDAC2 (unknown origin) using Ac-Leu-Gly-Lys(Ac)-AMC as substrate preincubated for 5 mins followed by substrate addition	[53]
	150	Inhibition of human HDAC2 after 40 mins by fluorescence analysis	[54]
	153	Inhibition of HDAC2 (unknown origin) after 30 mins by fluorescence assay	[55]
	159	Inhibition of HDAC1/2 in human HeLa cell nuclear extract using COLOR DE LYS as substrate pretreated for 5 mins followed by substrate addition	[55]
	161	Inhibition of HDAC1/2 in human HeLa cell nuclear extracts preincubated for 5 mins followed by substrate addition measured after 0.5 hrs by Color DE LYS assay	[56]
	164	Inhibition of human recombinant HDAC2	[57]
	164	Inhibition of full length C-terminal 6x-His tagged human HDAC2 using Arg-His-Lys-Lys(Ac) substrate incubated for 2 hrs by fluorescence assay	[58]
	164	The inhibitory effects of compounds on histone deacetylase (HDAC) activity were determined using a fluorescence-based assay	[59]
	170	Inhibition of HDAC2 (unknown origin)	[60]
	170	Inhibition of HDAC2 (unknown origin)	[61]
	170	Following transfection of HEK293 cells with pBJ5-HDAC1 wild type or mutant plasmids	[62]
	170	Inhibition of HDAC2	[63]
	174	Inhibition of recombinant C-terminal His-tagged full length human HDAC2 expressed in baculovirus infected insect Sf9 cells using fluorogenic HDAC substrate 3 after 30 mins by fluorescence assay	[64]
	175	Inhibition of HDAC2 (unknown origin)	[65]
	180	Inhibition of recombinant human HDAC2 preincubated with enzyme followed by flour de lys-green substrate addition measured after 1 hrs by fluorescence assay	[66]
	180	Inhibition of human recombinant HDAC2 using Fluor de Lys-Green as substrate preincubated for 5 mins followed by substrate addition and measured after 15 mins by fluorescence assay	[67]

HDAC2-inhibitors	IC_{50} (nM)	Methods of Determination	Reference
	180	Inhibition of recombinant HDAC2 using Ac-Lys(Ac)-AMC as substrate after 30 mins by fluorescence analysis	[68]
	182	Inhibition of human recombinant HDAC2 after 60 mins by fluorimetric assay	[69]
	185	Inhibition of HDAC2 (unknown origin) using Boc-Lys(acetyl)-AMC as substrate after 30 mins by fluorescence assay	[70]
	193	Inhibition of HDAC1/2 in human HeLa cells nuclear extracts using acetylated histone peptide as substrate after 30 mins by Color de Lys assay	[71]
	197	Inhibition of HDAC2 (unknown origin)	[72]
	200	Inhibition of human recombinant HDAC2 using Boc-L-Lys (Ac)-AMC as substrate preincubated with compound for 5 mins measured after 35 mins by spectrofluorometry assay	[4]
	200	Inhibition of full-length human recombinant C-terminal FLAG-tagged HDAC2 expressed in baculovirus infected Sf9 insect cells using Boc-Lys(epsilon-Ac)-AMC as fluorogenic substrate	[73]
	200	Inhibition of recombinant C-terminal FLAG-tagged HDAC2 expressed in baculovirus after 10 mins by fluorimetric analysis	[74]
	210	Inhibition of HDAC2 after 10 mins by fluorometric assay	[75]
	214	Inhibition of full length recombinant human HDAC2 expressed in baculovirus infected Sf9 insect cells using KI 177 as substrate preincubated for 5 min	[76]
	220	Inhibition of HDAC2 (unknown origin) using Boc-Lys(acetyl)-AMC as substrate preincubated for 5 mins followed by substrate addition measured after 30 mins	[49]
	220	Inhibition of HDAC2 (unknown origin) using fluorogenic Boc-Lys(acetyl)-AMC as substrate preincubated for 5 mins followed by substrate addition measure after 30 mins by fluorescence assay	[77]
	232	Inhibition of HDAC2 (unknown origin) using fluorogenic peptide from p53 residues (379 to 382) (RHKK(Ac)) as substrate by fluorescence assay	[78]
	240	Inhibition of human HDAC2 using ArgHisLysLys(Ac) fluorogenic peptide as a substrate by fluorimetric assay	[79]
	240	Inhibition of HDAC2 (unknown origin) using RHKK(Ac) fluorogenic acetylated peptide substrate by fluorometric assay	[80]
	240	Inhibition of HDAC2 (unknown origin) using fluorogenic peptide as substrate by fluorescence assay	[81]
	250	Inhibition of human recombinant HDAC2 by fluorometry	[82]
	251*	Biochemical assays of HDAC activity were carried out by Nanosyn in a reaction volume of 10 ul in 384-well microplates. A standard enzymatic reaction	[2]
	256	Inhibition of HDAC2 (unknown origin) using Boc-Lys(acetyl)-AMC as substrate preincubated with enzyme	[83]

HDAC2-inhibitors	IC_{50} (nM)	Methods of Determination	Reference
		for 5 mins prior to substrate addition measured after 30 mins by fluorescence assay	
	260	Inhibition of human recombinant HDAC2 using Boc-L-Lys (Ac)-AMC as substrate preincubated with compound for 24 hrs measured after 35 mins by spectrofluorometry assay	[4]
	280	Inhibition of recombinant HDAC2 (unknown origin) incubated for 10 mins using Boc-Lys(acetyl)-AMC fluorogenic substrate by homogeneous fluorescence release assay	[84]
	280	Inhibition of recombinant HDAC2 (unknown origin) using fluorogenic substrate Boc-Lys (acetyl)-AMC after 20 mins by homogeneous fluorescence release assay	[84]
	282	Inhibition of HDAC2 assessed as blockade of decorboxylation of carboxyfluorescein labeled acetylated peptide substrate after 17 hrs by fluorescence-based electrophoretic assay.	[85]
	282	Inhibition of HDAC2 (unknown origin) after 17 hrs	[86]
	282	Inhibition of human recombinant HDAC2	[87]
	333	Inhibition of recombinant human HDAC2 using fluorogenic substrate by fluorescence assay	[88]
	390	Inhibition of HDAC2	[89]
	400	Inhibition of HDAC2 by in vitro deacetylation assay	[90]
	410	Inhibition of HDAC2 (unknown origin) using RHKK(Ac) (379 to 382) p53 peptide as substrate by fluorescence assay	[91]
	416	Inhibition of HDAC1/HDAC2 in human HeLa cell extract incubated for 5 mins prior to substrate addition measured after 30 mins by microtitre plate reader	[92]
	454	Inhibition of HDAC 2 in human HeLa nuclear extract using HDAC substrate-3 measured after 60 mins by fluorometric analysis	[93]
	537	Inhibition of C-terminal His-tagged full length human recombinant HDAC2 expressed in baculovirus expression system using fluorogenic acetylated peptide as substrate	[94]
	550	Inhibition of HDAC2 (unknown origin) by fluorimetric assay	[95]
	683	Inhibition of HDAC2	US10011611 (2018)
	760	Inhibition of recombinant human HDAC2 after 60 mins by fluorescence assay	[96]
	780	Inhibition of HDAC2 by fluorometric assay	[97]
	920	Inhibition of human HDAC2 by fluorescence assay	[98]
	920	Inhibition of human HDAC-2 using RHKK(Ac) as substrate by fluorescence assay	[99]
	921	Inhibition of human HDAC2 using RHKK(Ac) as substrate by fluorimetric analysis	[100]
	921	Inhibition of human HDAC-2 using RHKK(Ac) as substrate addition	[101]

HDAC2-inhibitors	IC_{50} (nM)	Methods of Determination	Reference
	921	Inhibition of human HDAC2 using RHKK(Ac) as substrate by fluorimetric analysis	[102]
	921	Inhibition of human HDAC2 by fluorimetric assay	[103]
	921	Inhibition of HDAC2 (unknown origin) using fluorogenic tetrapeptide RHKK(Ac) substrate by fluorescence assay	[104]
	1,060	The HDAC enzymatic assay was performed using a Fluorogenic HDAC Assay Kit (BPS Bioscience) according to the manufacturer's instruction	[105]
	1,100	In vitro bioactivity evaluation of compounds 4a-n was performed by HDAC activity assays using a HDAC colorimetric activity assay kit	[106]
20Y (9 assays)	13	Recombinant HDAC2 assay (preincubated for 1 hrs, reaction 15 mins)	[9]
	13	Recombinant HDAC2 assay (preincubated for 1 hrs, reaction 15 mins)	[107]
	49	Recombinant HDAC2 assay (preincubated for 1 hrs, reaction 2.5 hrs)	[62]
	49	Inhibition of HDAC2	[5]
	56.3*	Recombinant HDAC2 assay (preincubated for 15 mins, reaction 3 hrs)	[2]
	70	Recombinant HDAC2 assay (incubated for 1.5 hrs, reaction 15 mins)	[108]
	100	Inhibition of HDAC2	[109]
	100	Inhibition of recombinant HDAC2	[110]
	100	Inhibition of HDAC2	[111]
IWX (1 assays)	62*	Recombinant HDAC2 assay (preincubated for 1 hrs, reaction 15 mins)	[107]
6EZ (1 assays)	168*	Recombinant HDAC2 assay (preincubated for 1 hrs, reaction 15 mins)	[107]

*****values selected for the use in our calculation, which are results from the most similar methods of determination.

References

1. Bressi JC, Jennings AJ, Skene R, Wu Y, Melkus R, De Jong R, et al. Exploration of the HDAC2 foot pocket: Synthesis and SAR of substituted N-(2-aminophenyl)benzamides. Bioorg Med Chem Lett. 2010;20(10):3142-5.
2. Lauffer BE, Mintzer R, Fong R, Mukund S, Tam C, Zilberleyb I, et al. Histone deacetylase (HDAC) inhibitor kinetic rate constants correlate with cellular histone acetylation but not transcription and cell viability. J Biol Chem. 2013;288(37):26926-43.
3. Wagner FF, Weïwer M, Steinbacher S, Schomburg A, Reinemer P, Gale JP, et al. Kinetic and structural insights into the binding of histone deacetylase 1 and 2 (HDAC1, 2) inhibitors. Bioorg Med Chem. 2016;24(18):4008-15.
4. Vaidya AS, Karumudi B, Mendonca E, Madriaga A, Abdelkarim H, van Breemen RB, et al. Design, synthesis, modeling, biological evaluation and

photoaffinity labeling studies of novel series of photoreactive benzamide probes for histone deacetylase 2. Bioorg Med Chem Lett. 2012;22(15):5025-30.
5. Methot JL, Chakravarty PK, Chenard M, Close J, Cruz JC, Dahlberg WK, et al. Exploration of the internal cavity of histone deacetylase (HDAC) with selective HDAC1/HDAC2 inhibitors (SHI-1:2). Bioorg Med Chem Lett. 2008;18(3):973-8.
6. Lai MJ, Huang HL, Pan SL, Liu YM, Peng CY, Lee HY, et al. Synthesis and biological evaluation of 1-arylsulfonyl-5-(N-hydroxyacrylamide)indoles as potent histone deacetylase inhibitors with antitumor activity in vivo. J Med Chem. 2012;55(8):3777-91.
7. Ghosh B, Zhao WN, Reis SA, Patnaik D, Fass DM, Tsai LH, et al. Dissecting structure-activity-relationships of crebinostat: Brain penetrant HDAC inhibitors for neuroepigenetic regulation. Bioorg Med Chem Lett. 2016;26(4):1265-71.
8. Frey RR, Wada CK, Garland RB, Curtin ML, Michaelides MR, Li J, et al. Trifluoromethyl ketones as inhibitors of histone deacetylase. Bioorg Med Chem Lett. 2002;12(23):3443-7.
9. Wagner FF, Lundh M, Kaya T, McCarren P, Zhang YL, Chattopadhyay S, et al. An Isochemogenic Set of Inhibitors To Define the Therapeutic Potential of Histone Deacetylases in β-Cell Protection. ACS Chem Biol. 2016;11(2):363-74.
10. Wagner FF, Olson DE, Gale JP, Kaya T, Weïwer M, Aidoud N, et al. Potent and selective inhibition of histone deacetylase 6 (HDAC6) does not require a surface-binding motif. J Med Chem. 2013;56(4):1772-6.
11. Jiao P, Jin P, Li C, Cui L, Dong L, Pan B, et al. Design, synthesis and in vitro evaluation of amidoximes as histone deacetylase inhibitors for cancer therapy. Bioorg Med Chem Lett. 2016;26(19):4679-83.
12. Hendricks JA, Keliher EJ, Marinelli B, Reiner T, Weissleder R, Mazitschek R. In vivo PET imaging of histone deacetylases by 18F-suberoylanilide hydroxamic acid (18F-SAHA). J Med Chem. 2011;54(15):5576-82.
13. Tan S, He F, Kong T, Wu J, Liu Z. Design, synthesis and tumor cell growth inhibitory activity of 3-nitro-2H-cheromene derivatives as histone deacetylaes inhibitors. Bioorg Med Chem. 2017;25(15):4123-32.
14. Moffat D, Patel S, Day F, Belfield A, Donald A, Rowlands M, et al. Discovery of 2-(6-{[(6-fluoroquinolin-2-yl)methyl]amino}bicyclo[3.1.0]hex-3-yl)-N-hydroxypyrimidine-5-carboxamide (CHR-3996), a class I selective orally active histone deacetylase inhibitor. J Med Chem. 2010;53(24):8663-78.
15. Tashima T, Murata H, Kodama H. Design and synthesis of novel and highly-active pan-histone deacetylase (pan-HDAC) inhibitors. Bioorg Med Chem. 2014;22(14):3720-31.

16. Duan YC, Ma YC, Qin WP, Ding LN, Zheng YC, Zhu YL, et al. Design and synthesis of tranylcypromine derivatives as novel LSD1/HDACs dual inhibitors for cancer treatment. Eur J Med Chem. 2017;140:392-402.
17. Yang J, Cheng G, Xu Q, Luan S, Wang S, Liu D, et al. Design, synthesis and biological evaluation of novel hydroxamic acid based histone deacetylase 6 selective inhibitors bearing phenylpyrazol scaffold as surface recognition motif. Bioorg Med Chem. 2018;26(8):1418-25.
18. Ramesh R, Reddy DS. Quest for Novel Chemical Entities through Incorporation of Silicon in Drug Scaffolds. J Med Chem. 2018;61(9):3779-98.
19. Yang Z, Wang T, Wang F, Niu T, Liu Z, Chen X, et al. Discovery of Selective Histone Deacetylase 6 Inhibitors Using the Quinazoline as the Cap for the Treatment of Cancer. J Med Chem. 2016;59(4):1455-70.
20. Estiu G, West N, Mazitschek R, Greenberg E, Bradner JE, Wiest O. On the inhibition of histone deacetylase 8. Bioorg Med Chem. 2010;18(11):4103-10.
21. Chen W, Dong G, Wu Y, Zhang W, Miao C, Sheng C. Dual NAMPT/HDAC Inhibitors as a New Strategy for Multitargeting Antitumor Drug Discovery. ACS Med Chem Lett. 2018;9(1):34-8.
22. Daniel KB, Sullivan ED, Chen Y, Chan JC, Jennings PA, Fierke CA, et al. Dual-Mode HDAC Prodrug for Covalent Modification and Subsequent Inhibitor Release. J Med Chem. 2015;58(11):4812-21.
23. Tang W, Luo T, Greenberg EF, Bradner JE, Schreiber SL. Discovery of histone deacetylase 8 selective inhibitors. Bioorg Med Chem Lett. 2011;21(9):2601-5.
24. Hutt DM, Olsen CA, Vickers CJ, Herman D, Chalfant M, Montero A, et al. Potential Agents for Treating Cystic Fibrosis: Cyclic Tetrapeptides that Restore Trafficking and Activity of ΔF508-CFTR. ACS Med Chem Lett. 2011;2(9):703-7.
25. Wu TY, Hassig C, Wu Y, Ding S, Schultz PG. Design, synthesis, and activity of HDAC inhibitors with a N-formyl hydroxylamine head group. Bioorg Med Chem Lett. 2004;14(2):449-53.
26. Aboeldahab AMA, Beshr EAM, Shoman ME, Rabea SM, Aly OM. Spirohydantoins and 1,2,4-triazole-3-carboxamide derivatives as inhibitors of histone deacetylase: Design, synthesis, and biological evaluation. Eur J Med Chem. 2018;146:79-92.
27. Liu R, Wang J, Tang W, Fang H. Design and synthesis of a new generation of substituted purine hydroxamate analogs as histone deacetylase inhibitors. Bioorg Med Chem. 2016;24(7):1446-54.
28. Jones P, Altamura S, De Francesco R, Paz OG, Kinzel O, Mesiti G, et al. A novel series of potent and selective ketone histone deacetylase inhibitors with antitumor activity in vivo. J Med Chem. 2008;51(8):2350-3.
29. Attenni B, Ontoria JM, Cruz JC, Rowley M, Schultz-Fademrecht C, Steinkühler C, et al. Histone deacetylase inhibitors with a primary amide zinc binding group

display antitumor activity in xenograft model. Bioorg Med Chem Lett. 2009;19(11):3081-4.

30. Kinzel O, Llauger-Bufi L, Pescatore G, Rowley M, Schultz-Fademrecht C, Monteagudo E, et al. Discovery of a potent class I selective ketone histone deacetylase inhibitor with antitumor activity in vivo and optimized pharmacokinetic properties. J Med Chem. 2009;52(11):3453-6.
31. Pescatore G, Kinzel O, Attenni B, Cecchetti O, Fiore F, Fonsi M, et al. Optimization of a series of potent and selective ketone histone deacetylase inhibitors. Bioorg Med Chem Lett. 2008;18(20):5528-32.
32. Pavlik CM, Wong CY, Ononye S, Lopez DD, Engene N, McPhail KL, et al. Santacruzamate A, a potent and selective histone deacetylase inhibitor from the Panamanian marine cyanobacterium cf. Symploca sp. J Nat Prod. 2013;76(11):2026-33.
33. Carradori S, Ortuso F, Petzer A, Bagetta D, De Monte C, Secci D, et al. Design, synthesis and biochemical evaluation of novel multi-target inhibitors as potential anti-Parkinson agents. Eur J Med Chem. 2018;143:1543-52.
34. Negmeldin AT, Knoff JR, Pflum MKH. The structural requirements of histone deacetylase inhibitors: C4-modified SAHA analogs display dual HDAC6/HDAC8 selectivity. Eur J Med Chem. 2018;143:1790-806.
35. Negmeldin AT, Pflum MKH. The structural requirements of histone deacetylase inhibitors: SAHA analogs modified at the C5 position display dual HDAC6/8 selectivity. Bioorg Med Chem Lett. 2017;27(15):3254-8.
36. Neelarapu R, Holzle DL, Velaparthi S, Bai H, Brunsteiner M, Blond SY, et al. Design, synthesis, docking, and biological evaluation of novel diazide-containing isoxazole- and pyrazole-based histone deacetylase probes. J Med Chem. 2011;54(13):4350-64.
37. Dallavalle S, Cincinelli R, Nannei R, Merlini L, Morini G, Penco S, et al. Design, synthesis, and evaluation of biphenyl-4-yl-acrylohydroxamic acid derivatives as histone deacetylase (HDAC) inhibitors. Eur J Med Chem. 2009;44(5):1900-12.
38. Cincinelli R, Zwick V, Musso L, Zuco V, De Cesare M, Zunino F, et al. Biphenyl-4-yl-acrylohydroxamic acids: Identification of a novel indolyl-substituted HDAC inhibitor with antitumor activity. Eur J Med Chem. 2016;112:99-105.
39. Cincinelli R, Musso L, Giannini G, Zuco V, De Cesare M, Zunino F, et al. Influence of the adamantyl moiety on the activity of biphenylacrylohydroxamic acid-based HDAC inhibitors. Eur J Med Chem. 2014;79:251-9.
40. Chen JB, Chern TR, Wei TT, Chen CC, Lin JH, Fang JM. Design and synthesis of dual-action inhibitors targeting histone deacetylases and 3-hydroxy-3-

methylglutaryl coenzyme A reductase for cancer treatment. J Med Chem. 2013;56(9):3645-55.
41. Duan W, Hou J, Chu X, Li X, Zhang J, Li J, et al. Synthesis and biological evaluation of novel histone deacetylases inhibitors with nitric oxide releasing activity. Bioorg Med Chem. 2015;23(15):4481-8.
42. Kachhadia V, Rajagopal S, Ponpandian T, Vignesh R, Anandhan K, Prabhu D, et al. Orally available stilbene derivatives as potent HDAC inhibitors with antiproliferative activities and antitumor effects in human tumor xenografts. Eur J Med Chem. 2016;108:274-86.
43. Mehndiratta S, Wang RS, Huang HL, Su CJ, Hsu CM, Wu YW, et al. 4-Indolyl-N-hydroxyphenylacrylamides as potent HDAC class I and IIB inhibitors in vitro and in vivo. Eur J Med Chem. 2017;134:13-23.
44. Lee HY, Chang CY, Su CJ, Huang HL, Mehndiratta S, Chao YH, et al. 2-(Phenylsulfonyl)quinoline N-hydroxyacrylamides as potent anticancer agents inhibiting histone deacetylase. Eur J Med Chem. 2016;122:92-101.
45. Ma C, Cao J, Liang X, Huang Y, Wu P, Li Y, et al. Novel leucine ureido derivatives as aminopeptidase N inhibitors. Design, synthesis and activity evaluation. Eur J Med Chem. 2016;108:21-7.
46. Duan W, Li J, Inks ES, Chou CJ, Jia Y, Chu X, et al. Design, synthesis, and antitumor evaluation of novel histone deacetylase inhibitors equipped with a phenylsulfonylfuroxan module as a nitric oxide donor. J Med Chem. 2015;58(10):4325-38.
47. Han L, Wang L, Hou X, Fu H, Song W, Tang W, et al. Design, synthesis and preliminary bioactivity studies of 1,2-dihydrobenzo[d]isothiazol-3-one-1,1-dioxide hydroxamic acid derivatives as novel histone deacetylase inhibitors. Bioorg Med Chem. 2014;22(5):1529-38.
48. Wen J, Bao Y, Niu Q, Yang J, Fan Y, Li J, et al. Identification of N-(6-mercaptohexyl)-3-(4-pyridyl)-1H-pyrazole-5-carboxamide and its disulfide prodrug as potent histone deacetylase inhibitors with in vitro and in vivo anti-tumor efficacy. Eur J Med Chem. 2016;109:350-9.
49. Zhao C, Zang J, Ding Q, Inks ES, Xu W, Chou CJ, et al. Discovery of meta-sulfamoyl N-hydroxybenzamides as HDAC8 selective inhibitors. Eur J Med Chem. 2018;150:282-91.
50. Lai JI, Leman LJ, Ku S, Vickers CJ, Olsen CA, Montero A, et al. Cyclic tetrapeptide HDAC inhibitors as potential therapeutics for spinal muscular atrophy: Screening with iPSC-derived neuronal cells. Bioorg Med Chem Lett. 2017;27(15):3289-93.
51. Dai Y, Guo Y, Curtin ML, Li J, Pease LJ, Guo J, et al. A novel series of histone deacetylase inhibitors incorporating hetero aromatic ring systems as connection units. Bioorg Med Chem Lett. 2003;13(21):3817-20.

52. Dai Y, Guo Y, Guo J, Pease LJ, Li J, Marcotte PA, et al. Indole amide hydroxamic acids as potent inhibitors of histone deacetylases. Bioorg Med Chem Lett. 2003;13(11):1897-901.
53. Dong G, Chen W, Wang X, Yang X, Xu T, Wang P, et al. Small Molecule Inhibitors Simultaneously Targeting Cancer Metabolism and Epigenetics: Discovery of Novel Nicotinamide Phosphoribosyltransferase (NAMPT) and Histone Deacetylase (HDAC) Dual Inhibitors. J Med Chem. 2017;60(19):7965-83.
54. Musso L, Cincinelli R, Zuco V, Zunino F, Nurisso A, Cuendet M, et al. Investigation on the ZBG-functionality of phenyl-4-yl-acrylohydroxamic acid derivatives as histone deacetylase inhibitors. Bioorg Med Chem Lett. 2015;25(20):4457-60.
55. He S, Dong G, Wu S, Fang K, Miao Z, Wang W, et al. Small Molecules Simultaneously Inhibiting p53-Murine Double Minute 2 (MDM2) Interaction and Histone Deacetylases (HDACs): Discovery of Novel Multitargeting Antitumor Agents. J Med Chem. 2018;61(16):7245-60.
56. Wang L, Hou X, Fu H, Pan X, Xu W, Tang W, et al. Design, synthesis and preliminary bioactivity evaluations of substituted quinoline hydroxamic acid derivatives as novel histone deacetylase (HDAC) inhibitors. Bioorg Med Chem. 2015;23(15):4364-74.
57. Andrianov V, Gailite V, Lola D, Loza E, Semenikhina V, Kalvinsh I, et al. Novel amide derivatives as inhibitors of histone deacetylase: design, synthesis and SAR. Eur J Med Chem. 2009;44(3):1067-85.
58. Blackburn C, Barrett C, Brunson M, Chin J, England D, Garcia K, et al. Histone deacetylase inhibitors derived from 1,2,3,4-tetrahydropyrrolo[1,2-a]pyrazine and related heterocycles selective for the HDAC6 isoform. Bioorg Med Chem Lett. 2014;24(23):5450-4.
59. Chen Y, Lopez-Sanchez M, Savoy DN, Billadeau DD, Dow GS, Kozikowski AP. A series of potent and selective, triazolylphenyl-based histone deacetylases inhibitors with activity against pancreatic cancer cells and Plasmodium falciparum. J Med Chem. 2008;51(12):3437-48.
60. Rabal O, Sánchez-Arias JA, Cuadrado-Tejedor M, de Miguel I, Pérez-González M, García-Barroso C, et al. Design, Synthesis, and Biological Evaluation of First-in-Class Dual Acting Histone Deacetylases (HDACs) and Phosphodiesterase 5 (PDE5) Inhibitors for the Treatment of Alzheimer's Disease. J Med Chem. 2016;59(19):8967-9004.
61. Rabal O, Sánchez-Arias JA, Cuadrado-Tejedor M, de Miguel I, Pérez-González M, García-Barroso C, et al. Design, synthesis, biological evaluation and in vivo testing of dual phosphodiesterase 5 (PDE5) and histone deacetylase 6

(HDAC6)-selective inhibitors for the treatment of Alzheimer's disease. Eur J Med Chem. 2018;150:506-24.
62. Nalawansha DA, Pflum MK. LSD1 Substrate Binding and Gene Expression Are Affected by HDAC1-Mediated Deacetylation. ACS Chem Biol. 2017;12(1):254-64.
63. Witter DJ, Harrington P, Wilson KJ, Chenard M, Fleming JC, Haines B, et al. Optimization of biaryl Selective HDAC1&2 Inhibitors (SHI-1:2). Bioorg Med Chem Lett. 2008;18(2):726-31.
64. He S, Dong G, Wang Z, Chen W, Huang Y, Li Z, et al. Discovery of Novel Multiacting Topoisomerase I/II and Histone Deacetylase Inhibitors. ACS Med Chem Lett. 2015;6(3):239-43.
65. Li X, Hou J, Jiang Y, Liu X, Mu W, Jin Y, et al. Development of 3-hydroxycinnamamide-based HDAC inhibitors with potent in vitro and in vivo anti-tumor activity. Eur J Med Chem. 2015;89:628-37.
66. Xie R, Li Y, Tang P, Yuan Q. Rational design, synthesis and preliminary antitumor activity evaluation of a chlorambucil derivative with potent DNA/HDAC dual-targeting inhibitory activity. Bioorg Med Chem Lett. 2017;27(18):4415-20.
67. Xie R, Li Y, Tang P, Yuan Q. Design, synthesis and biological evaluation of novel 2-aminobenzamides containing dithiocarbamate moiety as histone deacetylase inhibitors and potent antitumor agents. Eur J Med Chem. 2018;143:320-33.
68. Vickers CJ, Olsen CA, Leman LJ, Ghadiri MR. Discovery of HDAC Inhibitors That Lack an Active Site Zn(2+)-Binding Functional Group. ACS Med Chem Lett. 2012;3(6):505-8.
69. Yao Y, Liao C, Li Z, Wang Z, Sun Q, Liu C, et al. Design, synthesis, and biological evaluation of 1, 3-disubstituted-pyrazole derivatives as new class I and IIb histone deacetylase inhibitors. Eur J Med Chem. 2014;86:639-52.
70. Li X, Inks ES, Hou J, Chou CJ, Zhang J, Jiang Y, et al. Discovery of the first N-hydroxycinnamamide-based histone deacetylase 1/3 dual inhibitors with potent oral antitumor activity. J Med Chem. 2014;57(8):3324-41.
71. Fu H, Han L, Hou X, Dun Y, Wang L, Gong X, et al. Design, synthesis and biological evaluation of saccharin-based N-hydroxybenzamides as histone deacetylases (HDACs) inhibitors. Bioorg Med Chem. 2015;23(17):5774-81.
72. Thaler F, Moretti L, Amici R, Abate A, Colombo A, Carenzi G, et al. Synthesis, biological characterization and molecular modeling insights of spirochromanes as potent HDAC inhibitors. Eur J Med Chem. 2016;108:53-67.
73. Krieger V, Hamacher A, Gertzen CGW, Senger J, Zwinderman MRH, Marek M, et al. Design, Multicomponent Synthesis, and Anticancer Activity of a Focused Histone Deacetylase (HDAC) Inhibitor Library with Peptoid-Based Cap Groups. J Med Chem. 2017;60(13):5493-506.

74. Tessier P, Smil DV, Wahhab A, Leit S, Rahil J, Li Z, et al. Diphenylmethylene hydroxamic acids as selective class IIa histone deacetylase inhibitors. Bioorg Med Chem Lett. 2009;19(19):5684-8.
75. Lamblin M, Dabbas B, Spingarn R, Mendoza-Sanchez R, Wang TT, An BS, et al. Vitamin D receptor agonist/histone deacetylase inhibitor molecular hybrids. Bioorg Med Chem. 2010;18(11):4119-37.
76. Chao SW, Chen LC, Yu CC, Liu CY, Lin TE, Guh JH, et al. Discovery of aliphatic-chain hydroxamates containing indole derivatives with potent class I histone deacetylase inhibitory activities. Eur J Med Chem. 2018;143:792-805.
77. Zang J, Liang X, Huang Y, Jia Y, Li X, Xu W, et al. Discovery of Novel Pazopanib-Based HDAC and VEGFR Dual Inhibitors Targeting Cancer Epigenetics and Angiogenesis Simultaneously. J Med Chem. 2018;61(12):5304-22.
78. Ko KS, Steffey ME, Brandvold KR, Soellner MB. Development of a chimeric c-Src kinase and HDAC inhibitor. ACS Med Chem Lett. 2013;4(8):779-83.
79. Botta CB, Cabri W, Cini E, De Cesare L, Fattorusso C, Giannini G, et al. Oxime amides as a novel zinc binding group in histone deacetylase inhibitors: synthesis, biological activity, and computational evaluation. J Med Chem. 2011;54(7):2165-82.
80. Muthyala R, Shin WS, Xie J, Sham YY. Discovery of 1-hydroxypyridine-2-thiones as selective histone deacetylase inhibitors and their potential application for treating leukemia. Bioorg Med Chem Lett. 2015;25(19):4320-4.
81. Tang G, Wong JC, Zhang W, Wang Z, Zhang N, Peng Z, et al. Identification of a novel aminotetralin class of HDAC6 and HDAC8 selective inhibitors. J Med Chem. 2014;57(19):8026-34.
82. Suzuki N, Suzuki T, Ota Y, Nakano T, Kurihara M, Okuda H, et al. Design, synthesis, and biological activity of boronic acid-based histone deacetylase inhibitors. J Med Chem. 2009;52(9):2909-22.
83. Zhang Y, Yang P, Chou CJ, Liu C, Wang X, Xu W. Development of. ACS Med Chem Lett. 2013;4(2):235-8.
84. Cai J, Wei H, Hong KH, Wu X, Zong X, Cao M, et al. Discovery, bioactivity and docking simulation of Vorinostat analogues containing 1,2,4-oxadiazole moiety as potent histone deacetylase inhibitors and antitumor agents. Bioorg Med Chem. 2015;23(13):3457-71.
85. He R, Chen Y, Ougolkov AV, Zhang JS, Savoy DN, Billadeau DD, et al. Synthesis and biological evaluation of triazol-4-ylphenyl-bearing histone deacetylase inhibitors as anticancer agents. J Med Chem. 2010;53(3):1347-56.
86. Kozikowski AP, Tapadar S, Luchini DN, Kim KH, Billadeau DD. Use of the nitrile oxide cycloaddition (NOC) reaction for molecular probe generation: a

new class of enzyme selective histone deacetylase inhibitors (HDACIs) showing picomolar activity at HDAC6. J Med Chem. 2008;51(15):4370-3.
87. Tapadar S, He R, Luchini DN, Billadeau DD, Kozikowski AP. Isoxazole moiety in the linker region of HDAC inhibitors adjacent to the Zn-chelating group: effects on HDAC biology and antiproliferative activity. Bioorg Med Chem Lett. 2009;19(11):3023-6.
88. Lee HY, Lee JF, Kumar S, Wu YW, HuangFu WC, Lai MJ, et al. 3-Aroylindoles display antitumor activity in vitro and in vivo: Effects of N1-substituents on biological activity. Eur J Med Chem. 2017;125:1268-78.
89. Conti P, Tamborini L, Pinto A, Sola L, Ettari R, Mercurio C, et al. Design and synthesis of novel isoxazole-based HDAC inhibitors. Eur J Med Chem. 2010;45(9):4331-8.
90. Hutt DM, Herman D, Rodrigues AP, Noel S, Pilewski JM, Matteson J, et al. Reduced histone deacetylase 7 activity restores function to misfolded CFTR in cystic fibrosis. Nat Chem Biol. 2010;6(1):25-33.
91. Lee HY, Tsai AC, Chen MC, Shen PJ, Cheng YC, Kuo CC, et al. Azaindolylsulfonamides, with a more selective inhibitory effect on histone deacetylase 6 activity, exhibit antitumor activity in colorectal cancer HCT116 cells. J Med Chem. 2014;57(10):4009-22.
92. Guan P, Wang L, Hou X, Wan Y, Xu W, Tang W, et al. Improved antiproliferative activity of 1,3,4-thiadiazole-containing histone deacetylase (HDAC) inhibitors by introduction of the heteroaromatic surface recognition motif. Bioorg Med Chem. 2014;22(21):5766-75.
93. Ahmad M, Aga MA, Bhat JA, Kumar B, Rouf A, Capalash N, et al. Exploring Derivatives of Quinazoline Alkaloid l-Vasicine as Cap Groups in the Design and Biological Mechanistic Evaluation of Novel Antitumor Histone Deacetylase Inhibitors. J Med Chem. 2017;60(8):3484-97.
94. Li X, Tu Z, Li H, Liu C, Li Z, Sun Q, et al. Biological evaluation of new largazole analogues: alteration of macrocyclic scaffold with click chemistry. ACS Med Chem Lett. 2013;4(1):132-6.
95. Zwick V, Nurisso A, Simões-Pires C, Bouchet S, Martinet N, Lehotzky A, et al. Cross metathesis with hydroxamate and benzamide BOC-protected alkenes to access HDAC inhibitors and their biological evaluation highlighted intrinsic activity of BOC-protected dihydroxamates. Bioorg Med Chem Lett. 2016;26(1):154-9.
96. Yao Y, Tu Z, Liao C, Wang Z, Li S, Yao H, et al. Discovery of Novel Class I Histone Deacetylase Inhibitors with Promising in Vitro and in Vivo Antitumor Activities. J Med Chem. 2015;58(19):7672-80.
97. Suzuki T, Ota Y, Ri M, Bando M, Gotoh A, Itoh Y, et al. Rapid discovery of highly potent and selective inhibitors of histone deacetylase 8 using click chemistry to generate candidate libraries. J Med Chem. 2012;55(22):9562-75.

98. Marek L, Hamacher A, Hansen FK, Kuna K, Gohlke H, Kassack MU, et al. Histone deacetylase (HDAC) inhibitors with a novel connecting unit linker region reveal a selectivity profile for HDAC4 and HDAC5 with improved activity against chemoresistant cancer cells. J Med Chem. 2013;56(2):427-36.
99. Taddei M, Ferrini S, Giannotti L, Corsi M, Manetti F, Giannini G, et al. Synthesis and evaluation of new Hsp90 inhibitors based on a 1,4,5-trisubstituted 1,2,3-triazole scaffold. J Med Chem. 2014;57(6):2258-74.
100. Taddei M, Cini E, Giannotti L, Giannini G, Battistuzzi G, Vignola D, et al. Lactam based 7-amino suberoylamide hydroxamic acids as potent HDAC inhibitors. Bioorg Med Chem Lett. 2014;24(1):61-4.
101. Baruchello R, Simoni D, Marchetti P, Rondanin R, Mangiola S, Costantini C, et al. 4,5,6,7-Tetrahydro-isoxazolo-[4,5-c]-pyridines as a new class of cytotoxic Hsp90 inhibitors. Eur J Med Chem. 2014;76:53-60.
102. Giannini G, Battistuzzi G, Vignola D. Hydroxamic acid based histone deacetylase inhibitors with confirmed activity against the malaria parasite. Bioorg Med Chem Lett. 2015;25(3):459-61.
103. Auzzas L, Larsson A, Matera R, Baraldi A, Deschênes-Simard B, Giannini G, et al. Non-natural macrocyclic inhibitors of histone deacetylases: design, synthesis, and activity. J Med Chem. 2010;53(23):8387-99.
104. Giannini G, Vesci L, Battistuzzi G, Vignola D, Milazzo FM, Guglielmi MB, et al. ST7612AA1, a thioacetate-ω(γ-lactam carboxamide) derivative selected from a novel generation of oral HDAC inhibitors. J Med Chem. 2014;57(20):8358-77.
105. Huong TT, Dung DT, Huan NV, Cuong LV, Hai PT, Huong LT, et al. Novel N-hydroxybenzamides incorporating 2-oxoindoline with unexpected potent histone deacetylase inhibitory effects and antitumor cytotoxicity. Bioorg Chem. 2017;71:160-9.
106. Jiao J, Fang H, Wang X, Zhu H, Jiang L, Xu W. Synthesis of a novel series of benzylether-containing cinnamoyl derivatives as histone deacetylase inhibitors. J Enzyme Inhib Med Chem. 2010;25(1):132-8.
107. Wagner FF, Weïwer M, Steinbacher S, Schomburg A, Reinemer P, Gale JP, et al. Kinetic and structural insights into the binding of histone deacetylase 1 and 2 (HDAC1, 2) inhibitors. Bioorg Med Chem. 2016;24(18):4008-15.
108. Huang L, Lai WH, Zhu L, Li W, Wei L, Lee KH, et al. Elimination of HIV-1 Latently Infected Cells by Gnidimacrin and a Selective HDAC Inhibitor. ACS Med Chem Lett. 2018;9(3):268-73.
109. Paris M, Porcelloni M, Binaschi M, Fattori D. Histone deacetylase inhibitors: from bench to clinic. J Med Chem. 2008;51(6):1505-29.
110. Bertrand P. Inside HDAC with HDAC inhibitors. Eur J Med Chem. 2010;45(6):2095-116.

111. Moradei OM, Mallais TC, Frechette S, Paquin I, Tessier PE, Leit SM, et al. Novel aminophenyl benzamide-type histone deacetylase inhibitors with enhanced potency and selectivity. J Med Chem. 2007;50(23):5543-6.
